# Coming in hot: using emotional journey maps to examine parental perceptions associated with presentation of their child with fever to the emergency department in England

**DOI:** 10.1136/bmjpo-2025-003640

**Published:** 2025-09-12

**Authors:** Courtney Franklin, David Taylor-Robinson, Enitan D Carrol, Paul Moran, Bernie Carter

**Affiliations:** 1 Department of Public Health, Policy and Systems, University of Liverpool, Liverpool, UK; 2 Department of Clinical Infection, Microbiology and Immunology, University of Liverpool Institute of Infection Veterinary and Ecological Sciences, Neston, UK; 3 Department of Paediatric Infectious Diseases and Immunology, Alder Hey Children’s NHS Foundation Trust, Liverpool; 4 National Institute of Health Research Applied Research Collaboration (North West Coast), Lead, UK; 5 Edge Hill University, Ormskirk, UK

**Keywords:** Health services research, Child Health, Qualitative research

## Abstract

**Introduction:**

Paediatric emergency department (ED) attendances and admissions in England for fever are extremely common and are increasing, despite little evidence of increased risk and severity of fever-related presentations. Fever is a cause of great concern and anxiety for parents and carers, and these factors have a strong influence on decision-making across every step of a child’s journey through the healthcare system. There remains a gap in evidence investigating the emotional influences of parental health-seeking behaviours for fever.

**Objective:**

To explore the journeys taken by parents for children (0–18 years) with fever in England, from noticing a fever, to contacting primary care services, to ED attendance and subsequent discharge.

**Design:**

Qualitative design, using a novel emotional journey map approach.

**Participants:**

11 parents who had taken their febrile child to hospital (2015–2023).

**Methods:**

Emotional journey maps were co-produced with consenting parent participants during semi-structured Zoom interviews (2022–2023).

**Results:**

Parents’ anxiety, fear and uncertainty strongly influenced decision-making throughout their child’s healthcare journey. The use of the emotional component in the journey maps helped to clearly visualise the factors that influenced feelings of frustration and negative experiences. Lack of care continuity, unclear fever guidance and exclusion from decision-making led to mistrust and strained relationships with healthcare professionals. Attendance at the ED was driven by uncertainty about illness severity, conflicting medical advice, barriers to primary care and confusing safety-netting, highlighting key areas for intervention.

**Conclusions:**

Findings have significant potential to inform how and why parents seek support from different services and help to aid understanding of gaps in fever education and health services. These maps provide a powerful health service user experience tool and have significant potential to inform how and why parents seek different services and help aid understanding of gaps in fever education and health services.

**WHAT IS ALREADY KNOWN ON THIS TOPIC**

- Fever is one of the most common presenting problems for paediatric emergency department (ED) attendances and admissions in England.
- Parental reasons for ED attendance for paediatric fever are complex and multifaceted, with parental anxiety as an influential factor of ED attendance.
- Misconceptions surrounding fever have been shown to be detrimental to appropriate health service use.

**WHAT THIS STUDY ADDS**

- Parental anxiety, fear and uncertainty were strong emotional drivers of decision-making across every step of a child’s journey through the healthcare system.
- A trusting relationship between parents and doctors is necessary to understand the advice given.
- A lack of continuity of care resulted in frustration, mistrust in doctors and hypervigilance.

**HOW THIS STUDY MIGHT AFFECT RESEARCH, PRACTICE OR POLICY**

- Consideration of beneficial interventions across the whole parent journey is key to preventing parental mismanagement of fever, reducing parental anxiety and promoting confidence to correctly identify and manage their febrile child as well as reducing potentially avoidable hospital attendances.
- We illustrate how patient-reported data can be collected using creative methods.
- Good communication and relationship building between parents and doctors should be a priority, including coherent and standardised information (such as medical thresholds for fever) across health sectors and at each major touchpoint throughout a child’s healthcare journey.

## Introduction

 Fever is one of the most common presenting problems for paediatric emergency department (ED) attendances and admissions in England.^1^ These attendances are increasing despite little evidence of increased severity of disease.^2^ This rise could therefore reflect factors such as barriers to accessing primary care services or other out-of-hours care and the need for more support for parents dealing with fever.

For parents, fever (including febrile seizures) is a cause of great concern and anxiety due to the various conceptions and beliefs attributed to it, and this can have an impact on the management of childhood fever.^3 4^ Misconceptions of fever as an illness itself, rather than a presenting complaint, have shown to be detrimental to appropriate health service use, including premature health-seeking behaviour and out-of-hours service use, and antipyretic administration.^5^ Parents’ knowledge and behaviours towards their febrile child are largely influenced by emotional drivers, such as fever phobia.^6^ Parental anxiety has been associated with risk aversion and fear of a more serious illness.^7^

Research has explored the pathways of children with serious infectious illnesses and parental preferences in unscheduled paediatric healthcare and fever management.^8^,^11^ Findings show that parental uncertainty about illness severity leads to complex, non-linear care pathways^8^ and that organisational, professional and family factors influence admission timing.^9^ Nicholson *et al*^10^ showed that parents value timely access, expertise and continuity, and Leigh *et al*^11^ emphasised the importance of reassurance and communication. Nevertheless, there remains a gap concerning paediatric fever pathways in England, particularly regarding the emotional factors that influence parental help-seeking behaviours.

Journey maps offer a novel approach to visualising healthcare services from the patient’s perspective,^12^ providing a holistic depiction of their navigation through the system.

Emotional mapping has been used to understand sensitive topics, highlight the emotional impact of care and improve healthcare experiences by revealing parents’ perspectives.^13 14^ To address gaps in the use of emotional journey maps (EJMs) in health research and in understanding the pathways parents take when seeking emergency care for febrile children, this study employs EJMs to explore emotional drivers of parental health-seeking behaviours for their febrile child. This approach aims to identify barriers, inform policymakers and pinpoint opportunities for improving care and policy in febrile child management.

In phase 1 of this qualitative study,^15^ we used interviews to explore parental concerns regarding paediatric fever and decision-making leading to ED attendance. The reasons for emergency attendance for fever are complex and multifaceted.^15^ Parental anxiety has a strong influence on decision-making across every step of a child’s journey through the healthcare system. In phase 2, the focus was on understanding parental emotional influencers of parental decision-making as they traversed the healthcare system, from the point of noticing their child was febrile to the child’s discharge from hospital.^16^ This paper reports on phase 2 and focuses on the use of EJMs, otherwise known as experience mapping.^14^

## Methods

### Study design

A qualitative design using EJMs^14^ to augment audio-recorded semi-structured online (Zoom Video Communications) parent interviews was undertaken. The study followed the Consolidated Criteria for Reporting Qualitative Research guideline.^17^

### Patient and public involvement

A National Institute for Health Research Applied Research Collaborative Northwest Coast Public Advisor (PM) was involved throughout this study, providing invaluable insight into the design and interpretation of results.

### Participants

Parents of children (0–18 years) who attended an ED with a fever (January 2015–2023) in England could self-refer via recruitment adverts.^15^ Virtual adverts were shared online (Facebook and X (formerly Twitter)) and distributed to local schools and community centres. Convenience and venue-based sampling facilitated timely access of potential participants. Phase 1 aimed to recruit 10–15 parents to ensure adequate and rich data.^15^ Since some parents shared multiple experiences, the adequacy of the final sample size was evaluated throughout the interview process and was open to the researcher’s ongoing interpretation.

### Design of emotional journey maps

EJMs were selected to provide holistic insight into parents’ experiences, encouraging reflection of the potential stimuli which may have triggered an emotional response that led to ED attendance or other health service use.

At the end of their interview, the parent was shown a template EJM to aid discussion. It was designed specifically with the intention that the participant and interviewer would work together in personalising their journey map. A core aim was to help the researcher understand the whole journey that each parent took when navigating their child’s journey. Since the use of journey maps investigating healthcare services is extremely limited, we drew on techniques used in commercial settings where maps are commonly used to define customer needs, problems, engagement and experiences.

The template included touchpoints (encounters) that aimed to help parents to explore who they had consulted for advice, how helpful this was and how they felt at each part of the journey. The first touchpoint was ‘notice fever,’ where focus was on the environment and conditions surrounding the child at the ‘start’ of their febrile experience. There was then a natural progression of touchpoints associated with the first actions the parent took in managing the fever (eg, managing the fever at home, contacting family for advice and accessing a primary care health service). A touchpoint about the decision to attend the ED was also included, as this helped to identify those participants who self-referred and why and those who were directed to attend the ED by another healthcare professional (HCP). Further touchpoints included experiences associated with the hospital, including the transport they arrived in, the time between arrival and their child being seen and their child’s treatment and discharge. These touchpoints were considered valuable as the timing of admission to hospital for children with serious infectious illness has previously been investigated.^9^ It was neither expected that every parent’s journey would be the same nor that all touchpoints would apply to every parent.

**Figure 1:**
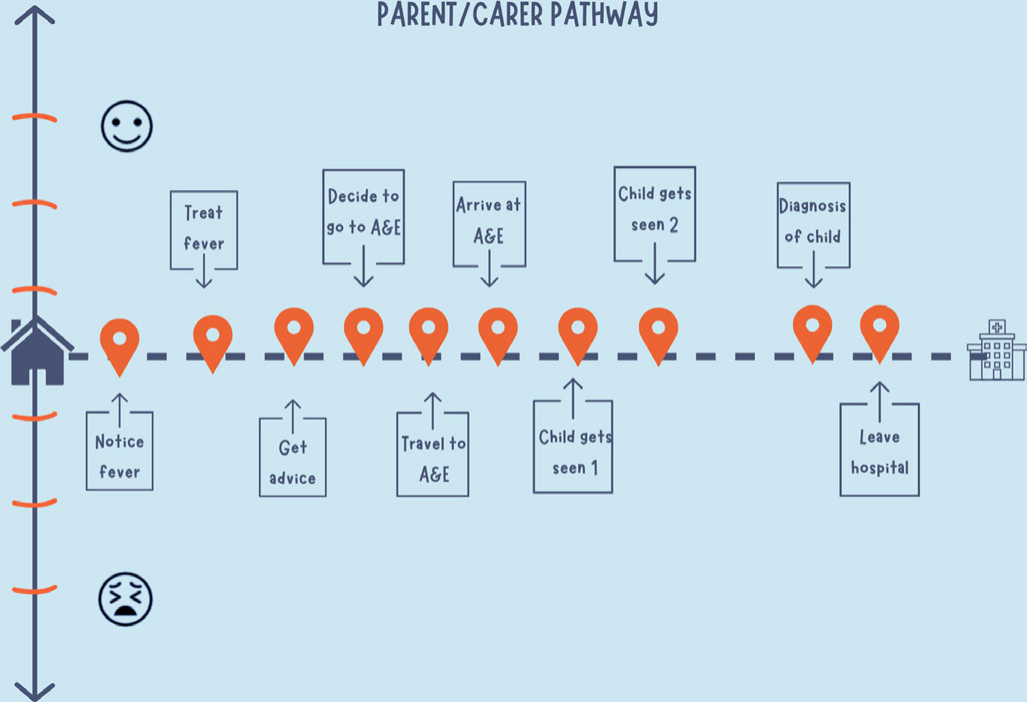
Example of the template used to map a parent journey shared during their interview. A&E, Accident & Emergency.

The timeline helped to unpick issues related to parental concern in terms of fever and its management, examining their relationship with their general practitioner (GP) and previous experience of presenting to GP and how seriously they felt their concerns were taken by HCPs. figure 1 shows an example of the template used to map a parent journey shared during their interview.

Emotional mapping using a six-point scale with visual end-point icons (happy face to anxious face) was incorporated to help depict the multidimensional relationship between the parent and the services that they navigated.^14^ This emotional scale aimed to visualise changes in parents’ concerns and/or anxieties and how this influenced their decision-making; it was not a validated tool and did not intend to generate objective validated data.

At the end of their interview, parents were invited to use the journey map template to add detail to their account of their experience seeking care for their febrile child, mapping their levels of anxiety or concern at each touchpoint. This activity was completed with the researcher, via screen-share. Parents identified that touchpoints they felt were most important to their whole experience. CF screen-shared the template and explained how to use the template, including explaining that each touchpoint could be changed and personalised depending on the participant’s own journey and that the emotional scale could be used as a guideline for tracking the participant’s level of concern/anxiety at each touchpoint. The latter was highlighted as subjective to how each participant interpreted the scale (eg, concern regarding their child’s condition or anxiety due to other external factors). Discussions during this exercise were audio-recorded. At the end of the EJM activity, CF had brief notes documented onto the parent’s map, plus the verbal explanation of what had happened and how they felt.

### Ethical considerations

Participants self-referred to the study and consent was gained before an appropriate time for an interview was scheduled. Anonymity was secured in the details and parent characteristics. Codes were used to ensure anonymity of participants: Mo, mother; Fa, father (followed by the participant number).

### Analysis

**Figure 2:**
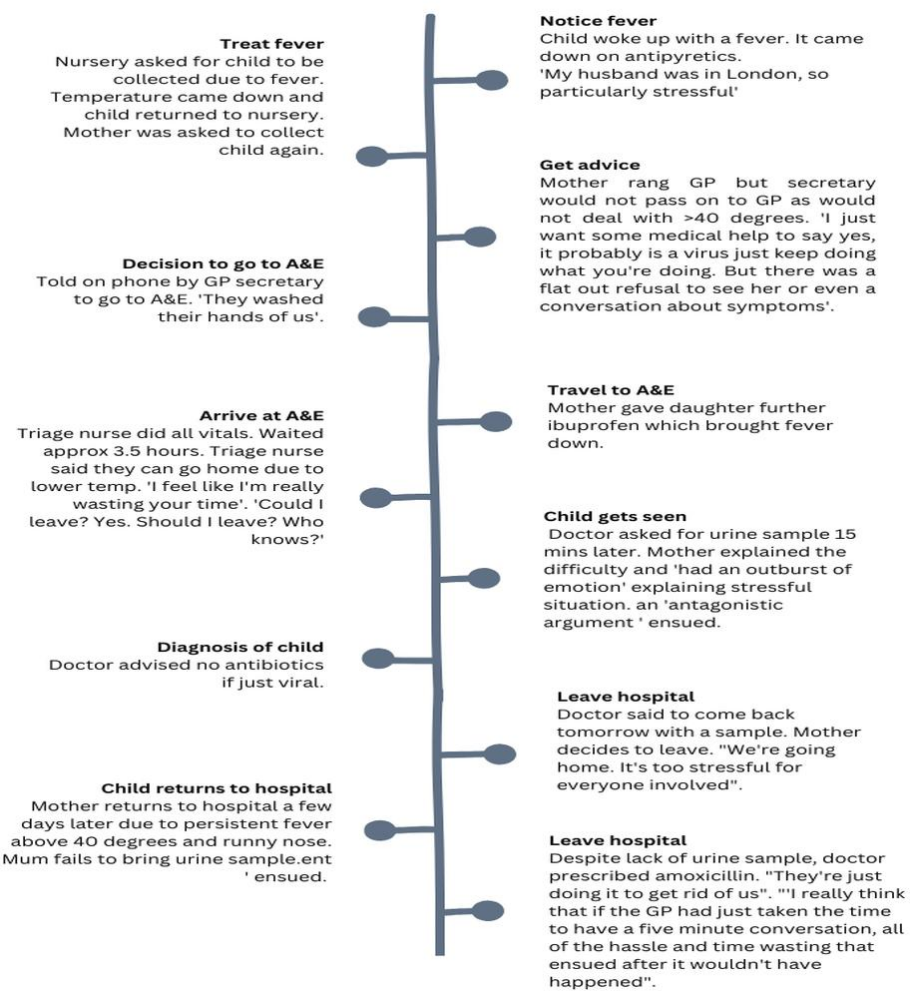
Example of parent journey touchpoints, Mo,3. GP, general practitioner; Mo, mother.

The initial notes created on each participant’s EJM were further developed using creative visual explorative approaches to generate an increasingly in-depth and structured journey map for each participant. For example, further context from the phase 1 interviews^15^ and conversations while building their maps was added to touchpoints, providing detail at each point on the EJM (figure 2).

EJMs are a relatively novel approach for use in health research, placing the individual at the centre of the research.^12 18 19^ Analysis of journey maps within healthcare is limited, and there is no specific recommended method for analysing journey maps. Therefore, a creative but robust approach was developed. However, techniques used in commercial settings^20^ proved useful in identifying patterns of repeated behaviours and potential triggers for such behaviours, as well as differentiating differences in the journeys, and highlighted potential problems associated with events. With this in mind, we focused on five domains—persona, activities, experience, problems and knowledge of fever and services (see table 1 for further details); these domains were added as new labels to the journey maps.

**Table 1:** Description of each domain included in emotional journey maps

Domain	Descriptor	Example
Persona	Represented the parent and their child. It detailed any perceived important descriptive information about the parent and child which could be relevant or influential to the parent’s personal experience.	Mother of daughter under 2 years living with husband. Both parents work full time. The daughter is in full-time nursery.
Activities	Summary of the events that occurred at each touchpoint and allowed further description of any key elements in the chronology of the parent’s recollection.	“Receptionists inform the parent to go to A&E as temperature is above 40” (analytical field note).
Experience	How each touchpoint made the parent feel to explore the rationale for their decision-making across their journey and allow direct exploration of between parents’ words (direct quotes) and the emotional scale scores.	“We’ve then got medical professionals with us so my worry has reduced” (Mo,4).
Problems	Identification of possible problems arising at each touchpoint and suggested resolution. This label used direct quotes, as well as analytical field notes.	“I do feel like had we been listened to properly, it would never have escalated as much as it did” (Mo,7). A need for clearer discharge information and safety netting to enable parents to better manage at home (analytical field note).
Knowledge of fever and services	Facilitated clear visualisation of each parent’s prior perceived proficiency and experience surrounding the management of fever. I decided this was also an important component of these journey maps, as thematic analysis revealed parental knowledge and proficiency in managing a fever as an important factor in parental decision-making. Therefore, this was considered just as important as the persona label, as it could influence the parent’s journey from the onset of their child’s fever. This was included as a touchpoint, or phase, on the map, rather than a label, as prior knowledge had its own activities, experiences and problems associated with it.	“It’s dealing with the receptionist staff that is the problem” (Fa,9).

Fa, father; Mo, mother.

In interpreting and presenting results, all journey maps were then grouped into broader categories: (1) a relatively calm journey with a period of extreme concern, (2) a journey about frustration and the need for reassurance and (3) a journey about an emergency ED visit (eg, febrile convulsion). This allowed further consideration of what public health interventions may be most beneficial and where they may be most effective. In the results, we present an example of categories 1 and 2, as these reflect the most typical example pathways.

## Results

### Participant characteristics

11 parents created a total of 15 EJMs, depicting their journey as they navigated care for their febrile child; of these, four parents shared two separate experiences (see online supplemental file 1 for participant characteristics). Recruitment was open between June 2022 and January 2023 after the minimum desired number of participants was recruited.

The journeys described were often complex when navigating health services for their febrile child, particularly when parents had negative experiences (eg, barriers accessing appropriate or preferred care); such experiences influenced feelings of frustration and concern.

10 parents initially contacted some form of primary care as a first step in gaining information or advice about their child’s fever before attending hospital. Advice came from the walk-in centre (n=4), pharmacy (n=1), their GP (n=4) or telephoned 111 (n=1) before attending hospital, indicating that they understood that these services were available and appropriate for their child at that time. Of these 10 experiences, nine hospital attendances were via primary care referrals and one was due to seeking out-of-hours care. Of the five experiences where parents did not contact primary care before hospital attendance, four were due to emergency symptoms.

All parents said their decision to present to the ED was a result of guidance or direct referral from other services. Of the 10 parents who contacted primary care services prior to hospital attendance, seven were referred directly to hospital, while for three parents, the decision to attend the ED was left to them. Four parents talked of experiences when their child was sent home from the ED prior to their reattendance. Of these, three parents still contacted primary care services before reattendance and were subsequently referred to hospital.

All parents identified themselves as relatively confident about home management of fever before the experience(s) presented in the EJMs. Of the 11 parents who gave an opinion of their relationship with the GP, three parents (three mothers) identified a positive relationship, four parents (three mothers and one father) identified a moderate relationship and four parents (three mothers and one father) identified a negative or no relationship.

Key drivers for heightened anxiety among parents included feeling they were not listened to, were stigmatised for being an inappropriate attender and feeling uncertainty surrounding medical advice and treatments. Despite most journey maps (n=12) concluding on a positive emotional scale (no anxiety—overall happy), most parents highlighted a need for better education and support surrounding symptoms and management of fever, especially febrile convulsions.

To illustrate the sorts of journeys taken by parents, two typical examples are presented: (1) an overall relatively calm journey with a period of extreme concern and (2) a journey characterised by parental frustration and the need for reassurance.

### A relatively calm journey with a period of extreme concern

**Figure 3:**
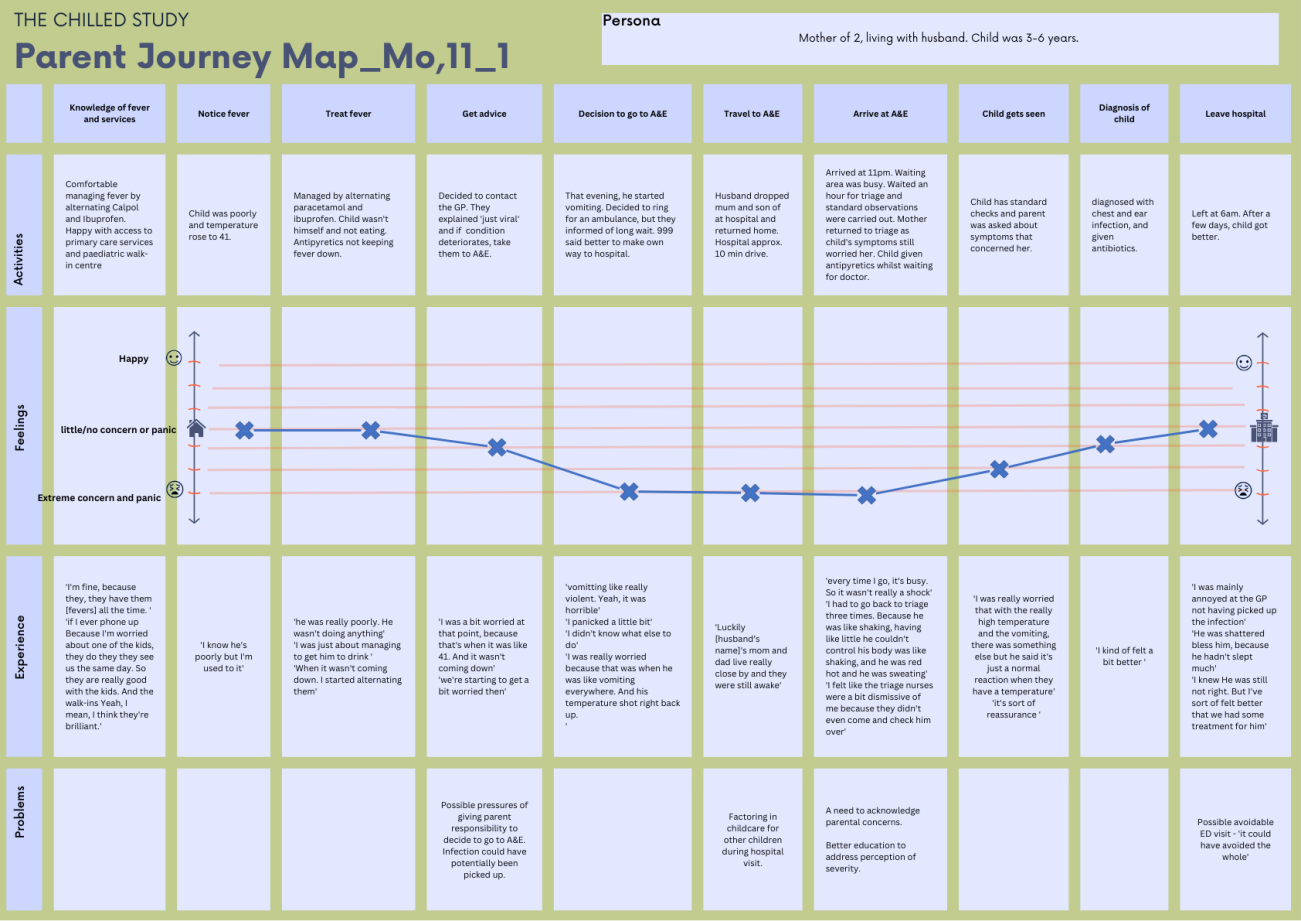
Journey map, Mo,11_01. Mo, mother.

Figure 3 presents the EJM of a mother of two children, living with her husband. Her son was 3–6 years old. This journey highlights two key drivers of emotional and practical concern: urgency to be seen and uncertainty surrounding her child’s condition.

Initially, this mother felt comfortable with home management of fever. She was aware of the health services accessible to her and had a good relationship with them, specifically identifying good access to a paediatric walk-in centre. At the onset of her child’s fever, she recalls measuring her child’s temperature (41°C); this triggered her to treat the fever by alternating ibuprofen and paracetamol. She indicated on the journey map she had little/no concern about the fever until her child displayed additional symptoms (not eating) and when she was unable to bring his temperature down. This resulted in her levels rising to ‘some concern’; at this point, she contacted the GP, explaining “she didn’t know what else to do.” The GP saw her child via in-person appointment, made a ‘just viral’ diagnosis and told her to present to the ED if his condition deteriorated. This juxtaposition of advice from the GP framing it as ‘just viral’ but advising ED attendance if additional symptoms developed raised concerns and seemingly shifted responsibility for her to make decisions.

That evening, her child experienced ‘violent vomiting’ where her concern levels rose to ‘extreme concern,’ and she dialled 999 for an ambulance. When told there was a long wait for an ambulance, her husband drove her and her son to the ED. The waiting room in the ED was ‘busy,’ and despite her son’s fever and additional symptoms, she felt dismissed by triage staff, heightening her concerns. When she was able to communicate her concerns to the ED doctor, her levels of concern dropped. She was relieved and reassured that her child’s symptoms were a ‘normal reaction to a temperature’ and that action had been taken. Her son’s diagnosis (chest and ear infection) and treatment (antibiotics) did not raise concern. At the end of her journey, she was no longer concerned. However, she expressed frustration towards the GP’s failure to diagnose the infection as this would have avoided the visit to the ED.

Overall, this EJM clearly shows the link between increases in parental concern and decision-making (eg, to get primary care advice and to attend the ED). Concerns remained until her son had been treated, was able to return home and she felt more in control again.

### A journey about frustration and the need for reassurance

**Figure 4:**
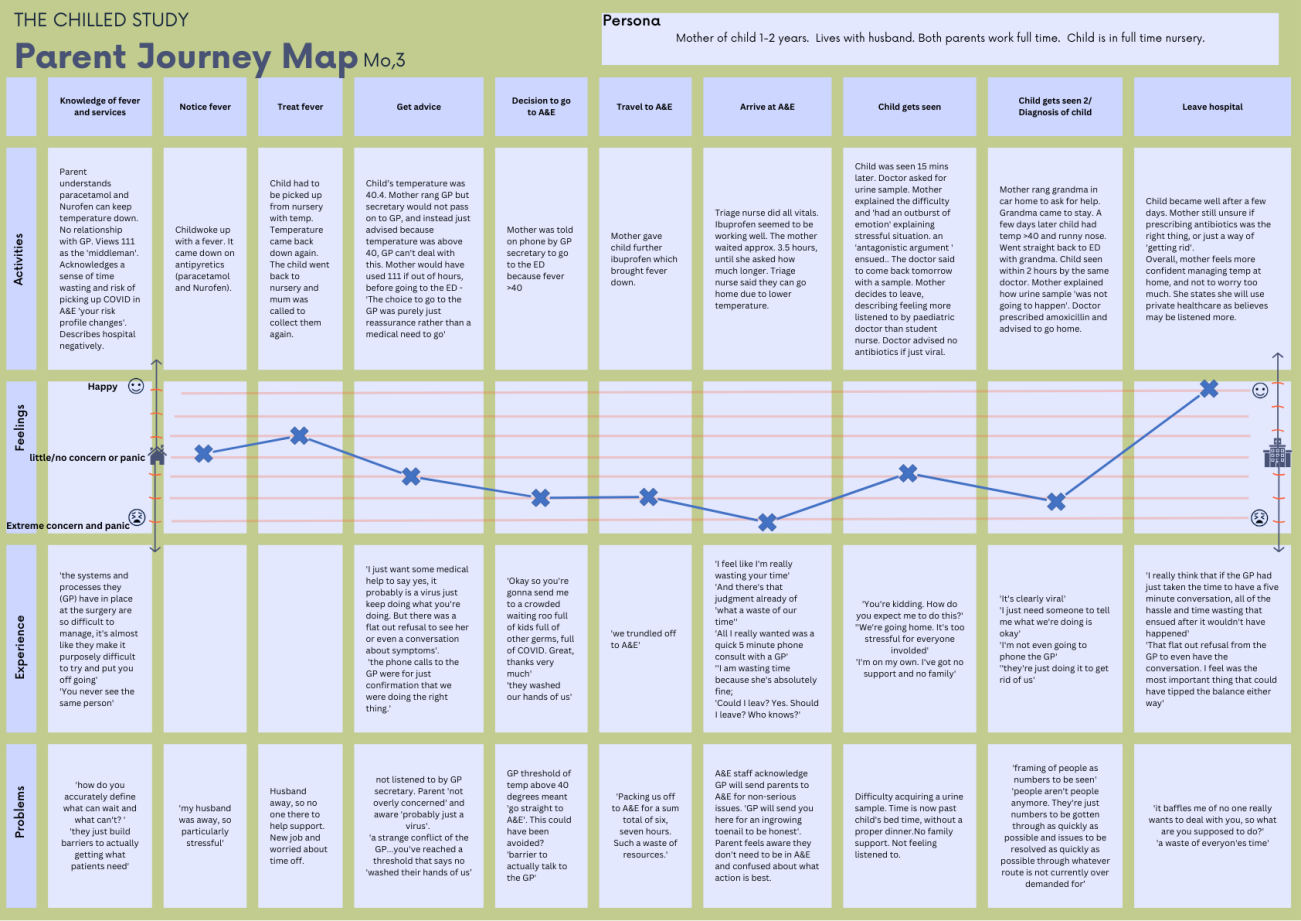
Journey map, Mo,3. ED, emergency department; GP; general practitioner.

Figure 4 presents the EJM of a mother and her daughter. Both parents worked full time, and her daughter (<2 years old) was in nursery while the parents were at work. This journey was illustrative of how the need for reassurance and barriers to and frustrations with health services and HCPs can be major emotional drivers of healthcare use.

This mother displayed good knowledge towards home management of fever and awareness of the variety of services she could access but talked of a negative relationship with her GP. At the onset of her child’s fever, she alternated paracetamol and ibuprofen and was successful in reducing the fever with no undue concern towards her daughter’s fever. However, after her daughter’s nursery had asked her to be picked up for two consecutive days, she rang the GP for reassurance, explaining she did not think she needed to attend the GP. On hearing that the child’s temperature was >40°C, the GP refused a consultation and advised she attend the ED. This triggered an acceleration of parental concern as she described feeling dismissed by the GP and was concerned that the ED would be ‘crowded’ and ‘full of other germs.’

Continuing to treat her daughter with antipyretics while waiting in the ED for over 3 hours, she was told by a triage nurse that her child’s temperature was low enough to return home. However, she decided to continue to wait to be seen by a doctor who, after examining her daughter, requested a urine sample. When the mother explained the difficulty of obtaining a sample from her daughter, the doctor proposed the source of the fever was viral, and antibiotics would not be prescribed.

After a few days at home, with the fever persisting (>40°C), her daughter developed a runny nose. Despite remaining confident the source of her child’s fever was ‘viral,’ she bypassed primary care and returned to the ED for reassurance. She saw the same ED doctor and was sent home with antibiotics despite no confirmation of bacterial infection. She left feeling dismissed.

The extreme concern and stress she experienced throughout most of her journey arose not from her daughter’s fever but from feeling stigmatised, frustrated and dismissed as an inappropriate hospital attender. Confusion arose in relation to differing thresholds for fever between her GP and the ED doctor and inconsistent decisions by the ED doctor about prescribing antibiotics in the absence of a diagnosis of bacterial infection. She experienced isolation as her husband was away much of the time her daughter was ill. These feelings were influential in the services she navigated, when and why she accessed them. Her experiences have the potential to influence her accessing services (especially her GP) in the future; she was clear she would no longer use NHS services preferring private healthcare.

## Discussion

To our knowledge, this is the first study to provide a holistic parent-centred perspective of care that characterised healthcare interactions across the whole journey with a febrile child. These findings complete a qualitative study investigating parental perceptions of reasons for ED presentation for paediatric fever in England.^15^ We expand the evidence base of using novel EJM approaches with parents to understand their febrile child’s journey to hospital and parents’ experiences of navigating healthcare services. We illustrate the complex, multi-faceted nature of the reasons for attending the ED, such as uncertainty surrounding disease severity, conflicting medical advice across health services, frustrations towards barriers accessing primary care and identifying areas for intervention entry points.

Parental anxiety has been linked to increased emergency care presentations and hospital admissions.^21^ We support existing evidence that anxieties regarding fever can initiate a fundamental change in parents’ management of their febrile child, including risk aversion when parents ‘err on the side of caution’ and have lower thresholds for seeking emergency help.^21 22^ Accompanying symptoms and the possible complications heightened parental fear.^23 24^ Our results also support findings that parents’ perceived vulnerability (including age) of their child often initiated parental health-seeking behaviour.^15^

ED attendance was a last resort for our parents despite the difficulties they experienced navigating a wide range of health services (eg, barriers to accessing GP, lack of familiarity or trust with HCPs), as reported elsewhere.^25^

Parental confidence was weaker among those who had received inconsistent information from HCPs.^7^ Previous studies have established an association between the loss of continuity in general practice and increased hospital admissions.^2^ We support findings that a trusting relationship between parents and doctors is necessary to understand the advice given.^26^ A lack of continuity of care resulted in frustration, mistrust in HCPs and hypervigilance.^27^ Both parents in the examples provided alternated antipyretics, which is only recommended by the National Institute for Health and Care Excellence as a reasonable option if monotherapy has been unsuccessful,^28^ further highlighting barriers in parents receiving the correct management recommendations.

‘Just viral’ or unspecific diagnosis made parents feel their concerns had been trivialised, as reported elsewhere.^23^ A lack of clear guidance on the warning signs of fever, or absence in involving parents in the decision-making process, provoked aggravated relationships with HCPs. This could cause further consequences such as decreased parental capacity to cope with home management of self-limiting illnesses and narrowed perceived access to health services.^29^ We support recent findings^30^ that parents desire clear verbal information from doctors and clear validation of their concerns.

A key strength of our novel work is the use of EJMs, which is mostly used within the commercial sector^12^ and increasingly being used in healthcare. We widen the evidence base showcasing how this method has the potential to identify gaps in health services, inform patient-centred, experience-based models of care^12 18^ and can aid the design of patient-centric solutions, incorporating the experiences and voices of health service users to improve health service quality.^31^ It aided deeper understanding of how parents of febrile children enter, experience and exit health services, with a particular focus on an ED setting. The additional emotional component helped explore how parents’ emotions influenced health-seeking behaviours. The graphical representations of the journeys presented in this study (figures2  3) extend existing ways of visualising journeys by both incorporating emotional responses and mapping our five domains: persona, activities, experience, problems and knowledge of fever and services.

The emotional component may introduce subjectivity, as each participant could interpret emotions on the map’s scale differently. However, this study focused on understanding the factors influencing changes in parents’ emotions and how these changes could impact their future healthcare decisions. Further limitations include sampling bias, small sample size, lack of socioeconomic data and potential social desirability bias. However, efforts were made to engage participants sensitively and to ensure the sample was heterogeneous in age, family structure, experience and healthcare use.^15^

Future research on paediatric fever, healthcare pathways and reducing unnecessary hospital attendance should examine differences in emotional factors and pathways across socioeconomic groups. Journey maps could offer insights into the influence of social determinants and empower participants to share rich data. Additionally, investigating specific communities, such as ethnic minorities and marginalised groups, could uncover intersectional factors in parental decision-making. Given the pandemic’s impact on parents’ awareness of infectious disease and healthcare access,^32 33^ repeating similar analyses post COVID would help assess whether these experiences influence current decision-making regarding children’s care.

These maps help to consider and identify intervention points to better support parents. Successful healthcare experiences and positive long-term outcomes are driven by good communication and relationship building with HCPs;^34^ these factors should be a priority.^26^ Our journey map analysis illustrates the importance of this at every interaction as parents navigate the healthcare system.

Providing clear information to parents, including risk of recurrence and how to manage them, and providing emotional support could reduce inappropriate use of healthcare and hospitalisations and associated costs.^35^ The demand for clear, widely distributed safety netting for parents to correctly identify and manage fever is recommended.^36^ Not only does clear and explicit advice help to improve health literacy, but it is key to preventing parental mismanagement of fever,^37^ reducing parental anxiety^38^ and promoting confidence and empowerment for parents to correctly identify and manage their febrile child^30^ as well as reducing potentially avoidable hospital attendances^16^ and workload.^39^

Health services need to work towards the elimination of conflicting information. This information must be coherent and standardised (such as medical thresholds for fever) across health sectors and each major touchpoint throughout a child’s healthcare journey.^35^

Community-based interventions have previously been recommended to improve paediatric urgent care.^40^ For example, holistic children’s ‘hubs’ supporting parental management of illnesses while also alleviating emergency health service workload could be more effective by further proportionate investment.

Findings from journey maps have significant potential to inform how and why parents seek support from different services and help to aid understanding of gaps in fever education and health services. Further, this approach contributes to the literature on how patient-reported data can be collected using creative methods. This method provided a holistic caregiver-centred perspective of care that characterised healthcare interactions across the whole patient journey and can be applied to wider public health objectives.

## Supplementary material

10.1136/bmjpo-2025-003640 online supplemental file 1

## Data Availability

All data relevant to the study are included in the article or uploaded as supplementary information.
