# Supplementary material for: Coming in hot: using emotional journey maps to examine parental perceptions associated with presentation of their child with fever to the emergency department in England
Source: BMJ Paediatr Open. 2025 Sep 12;9(1):e003640. doi: 10.1136/bmjpo-2025-003640 (PMC12519393; doi:10.1136/bmjpo-2025-003640)
Supplement: online supplemental file 1 [file bmjpo-9-1-s001.docx]

**SUPPLEMENTATY FILE**

**Parent characteristics**

| Participant | Participant’s gender | Location | First time parent | Number of child experiences discussed | Age range of child(ren)  (years) |
| --- | --- | --- | --- | --- | --- |
| Mo,3 | Female | Greater Manchester | Yes | 1 | 1-2 |
| Mo,4 | Female | Cheshire | Yes | 1 | 3-6 |
| Mo,5 | Female | Liverpool | No | 2 | 3-6 |
| Mo,6 | Female | Liverpool | No | 2 | <1 |
| Mo,7 | Female | Lancashire | Yes | 1 | 3-6 |
| Fa,8 | Male | Liverpool | No | 1 | 1-2 |
| Fa,9 | Male | Wirral | No | 1 | 3-6 |
| Mo,10 | Female | Liverpool | No | 1 | 3-6 |
| Mo,11 | Female | Liverpool | No | 2 | 3-6 |
| Mo,12 | Female | Lancashire | Yes | 2 | 1-2 |
| Mo,14 | Female | Liverpool | No | 1 | <1 |

Mo= mother, Fa= father

**Parent Emotional Journey Maps**


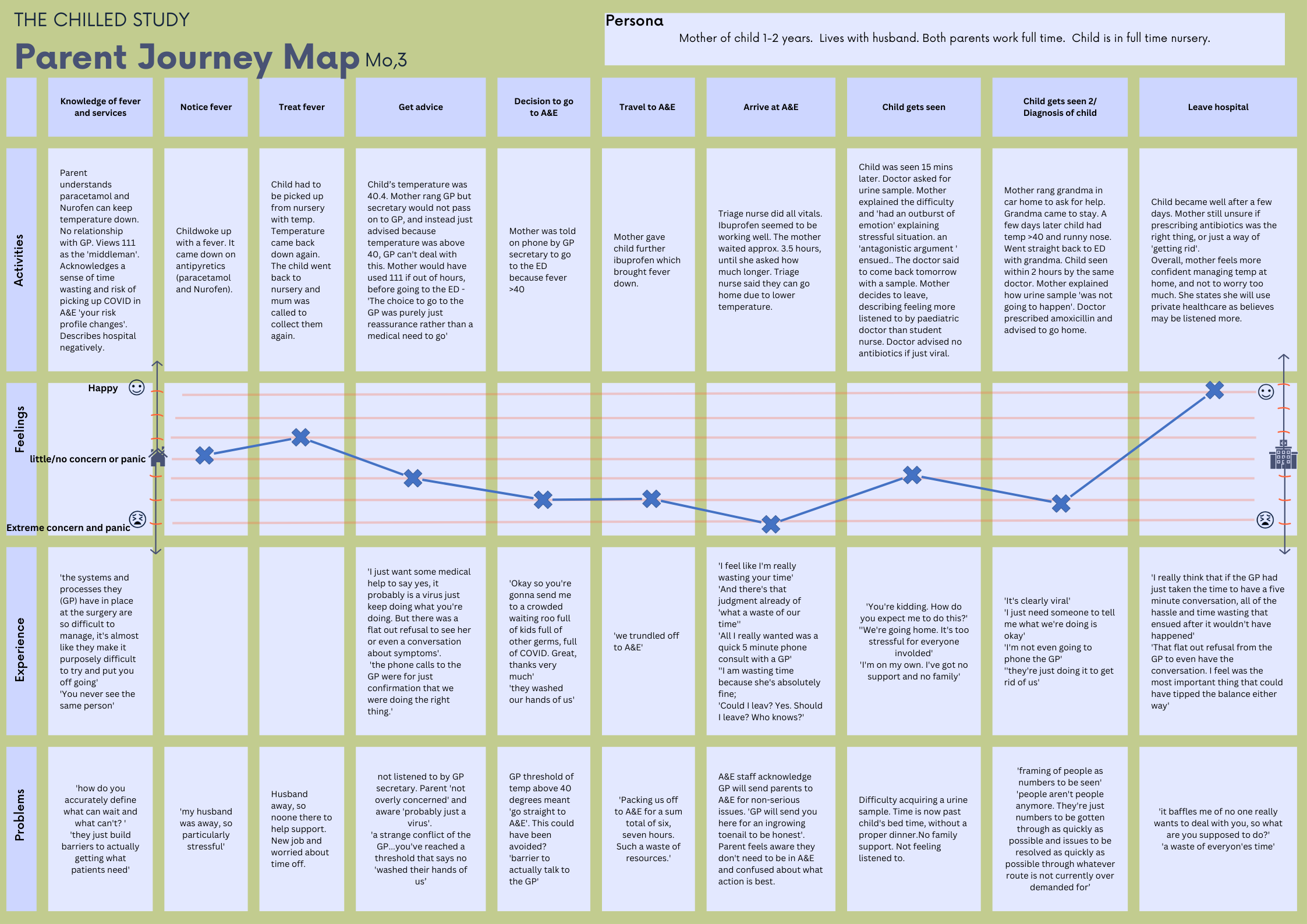


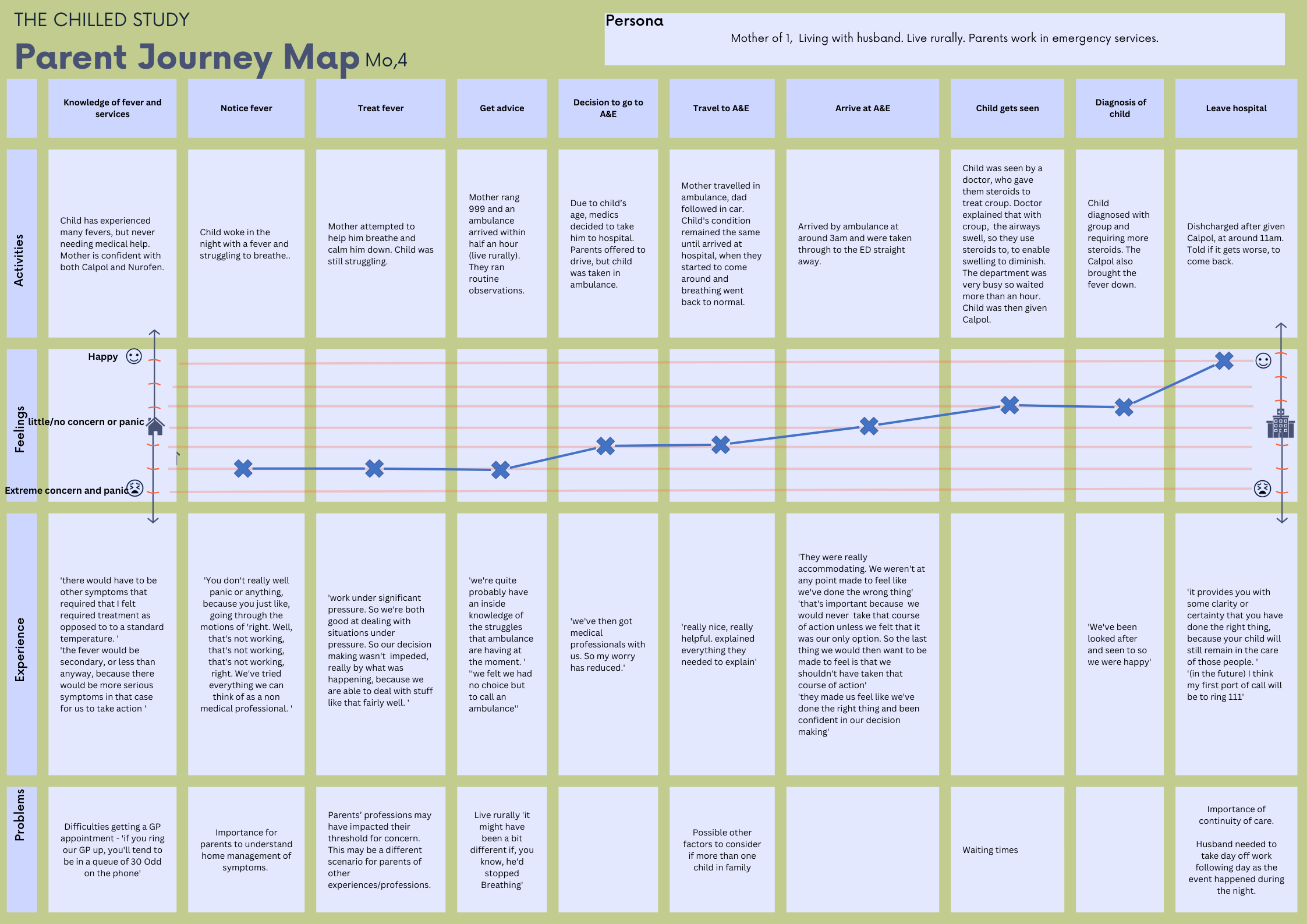

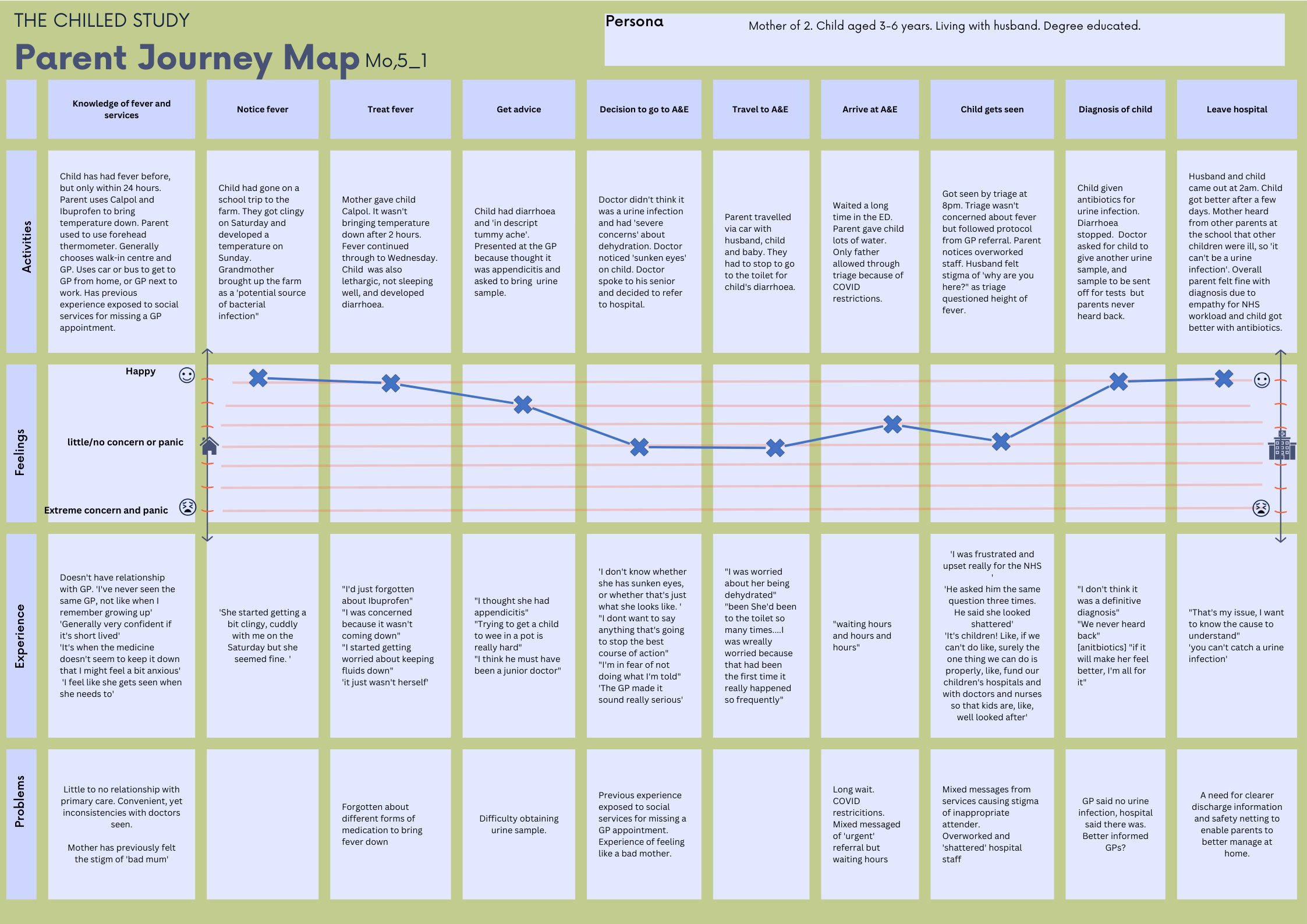

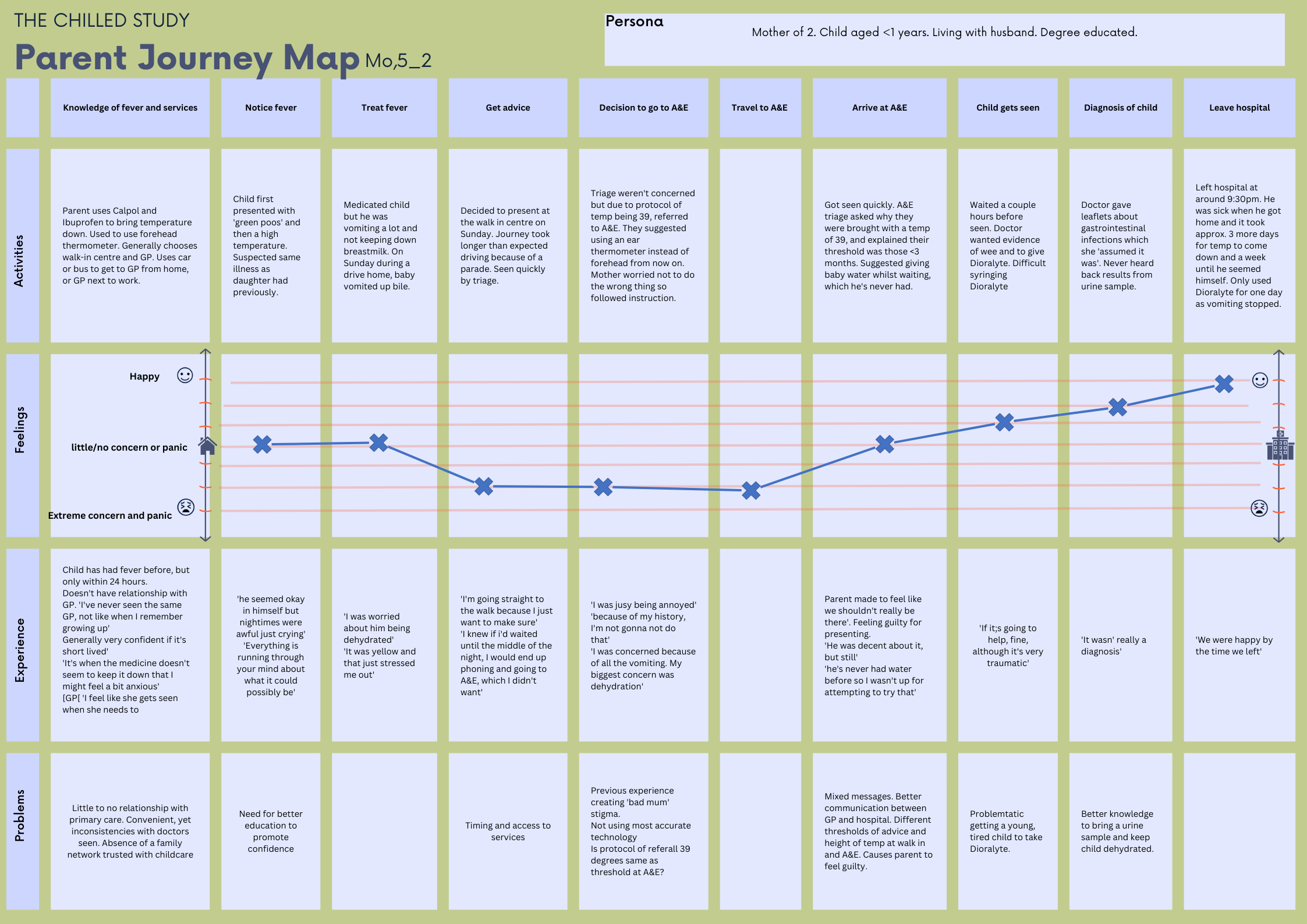

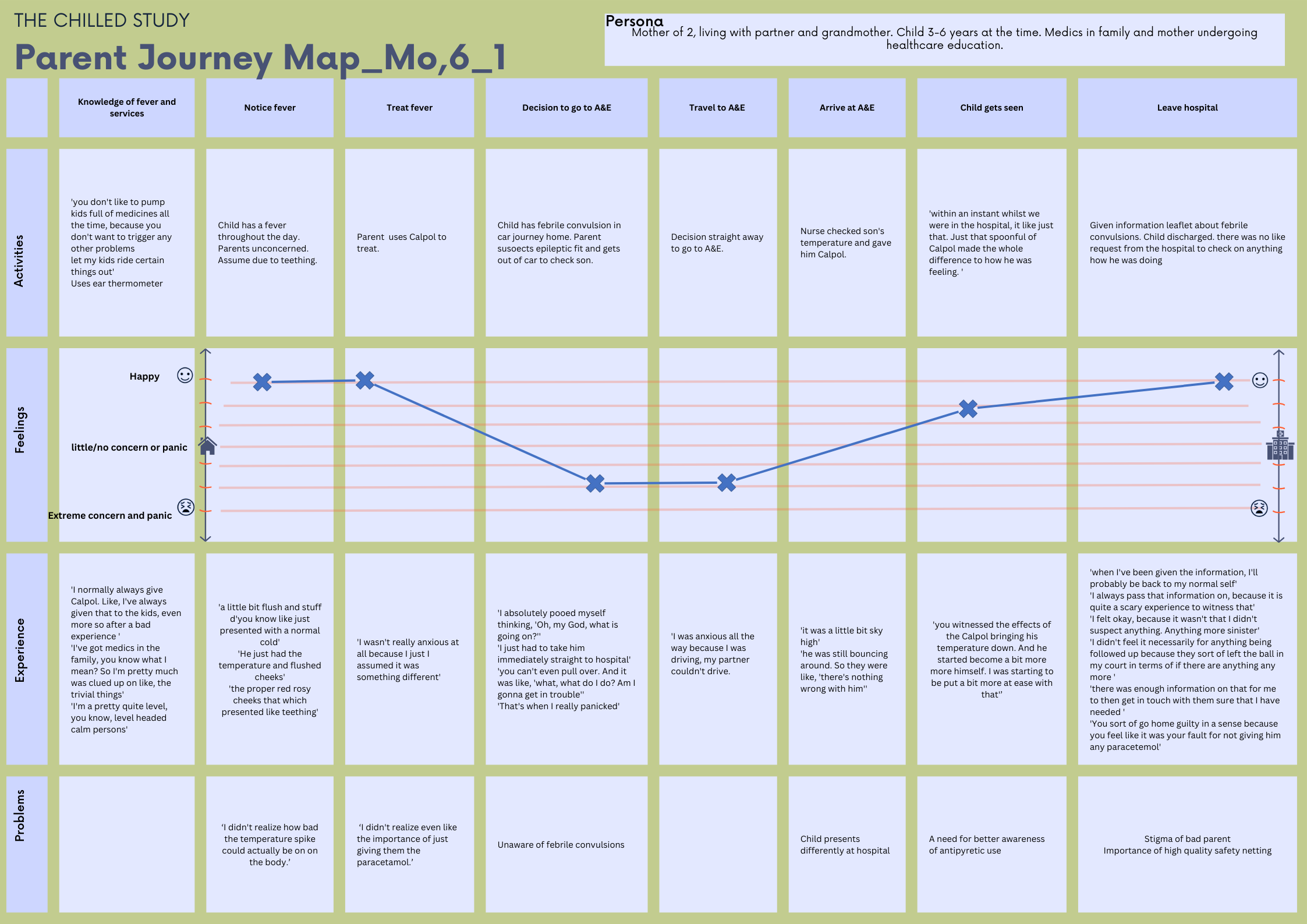

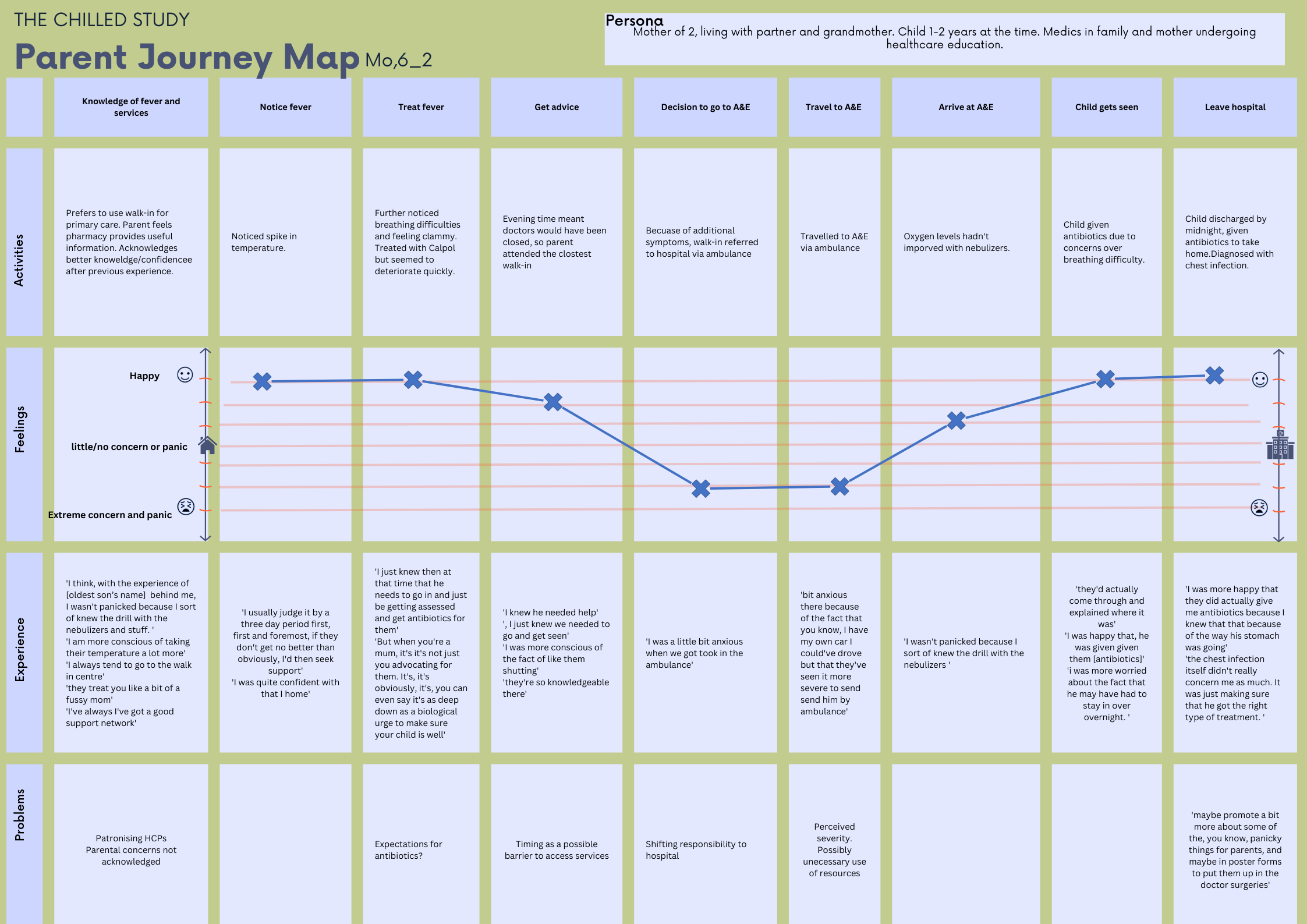

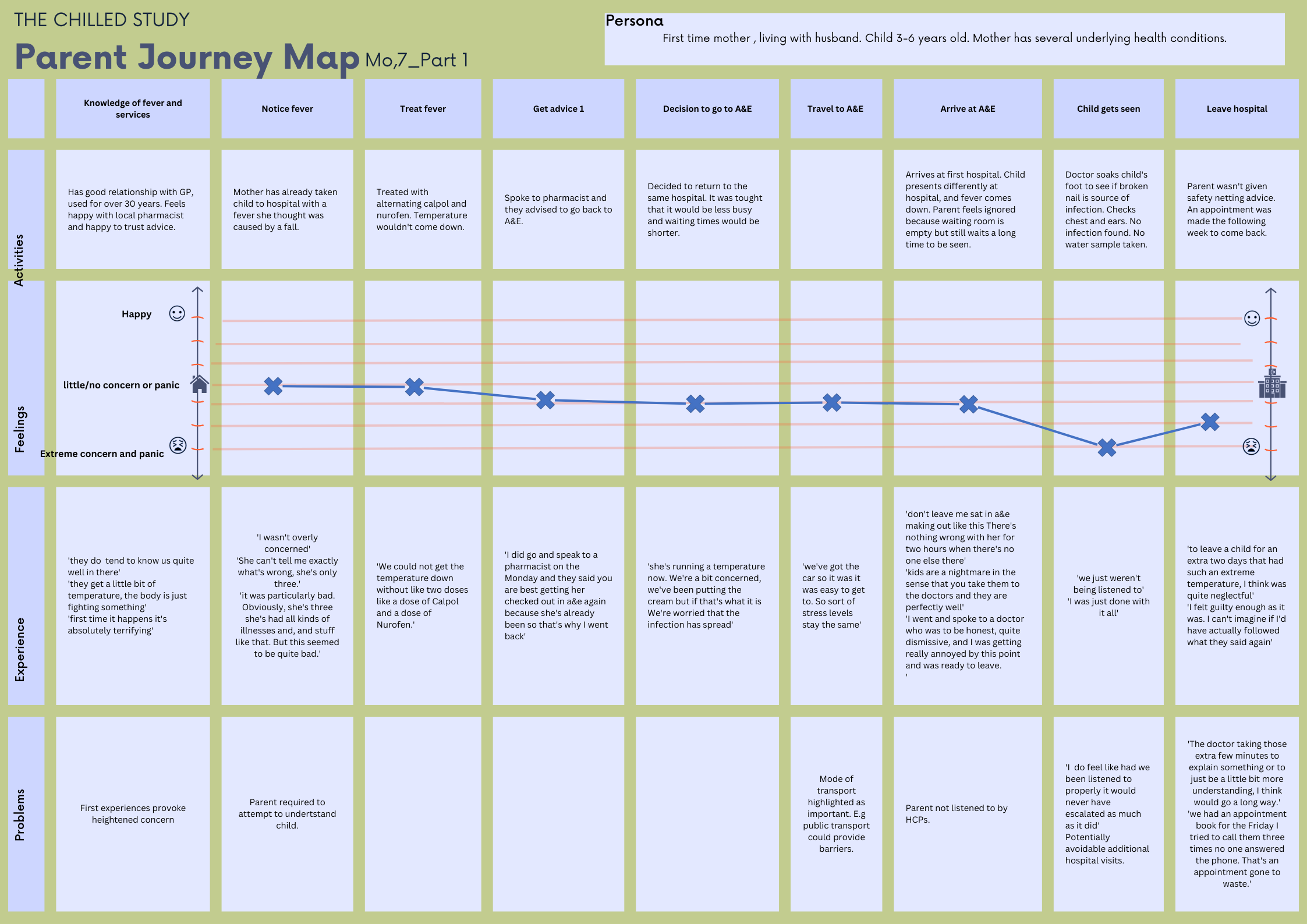


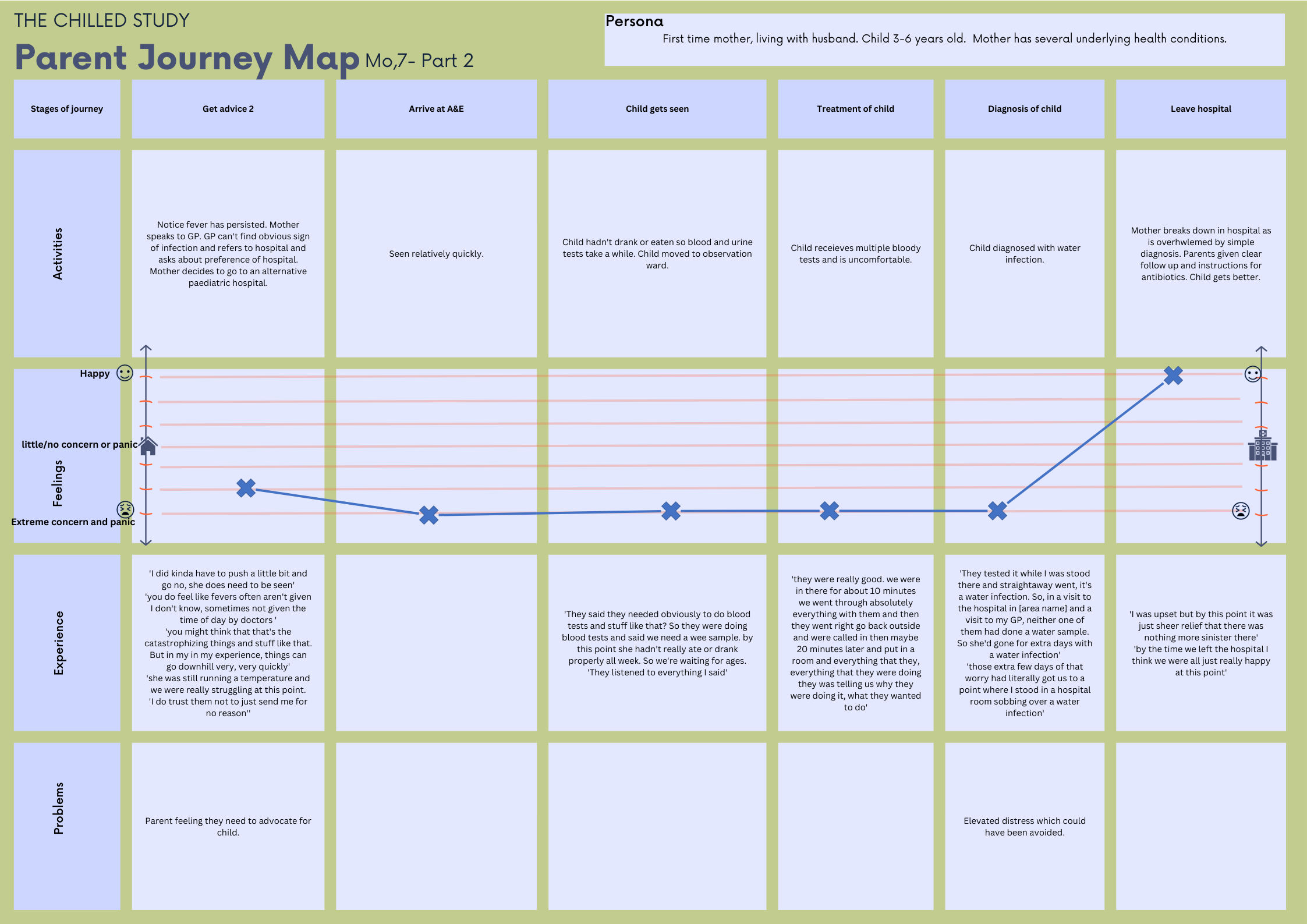

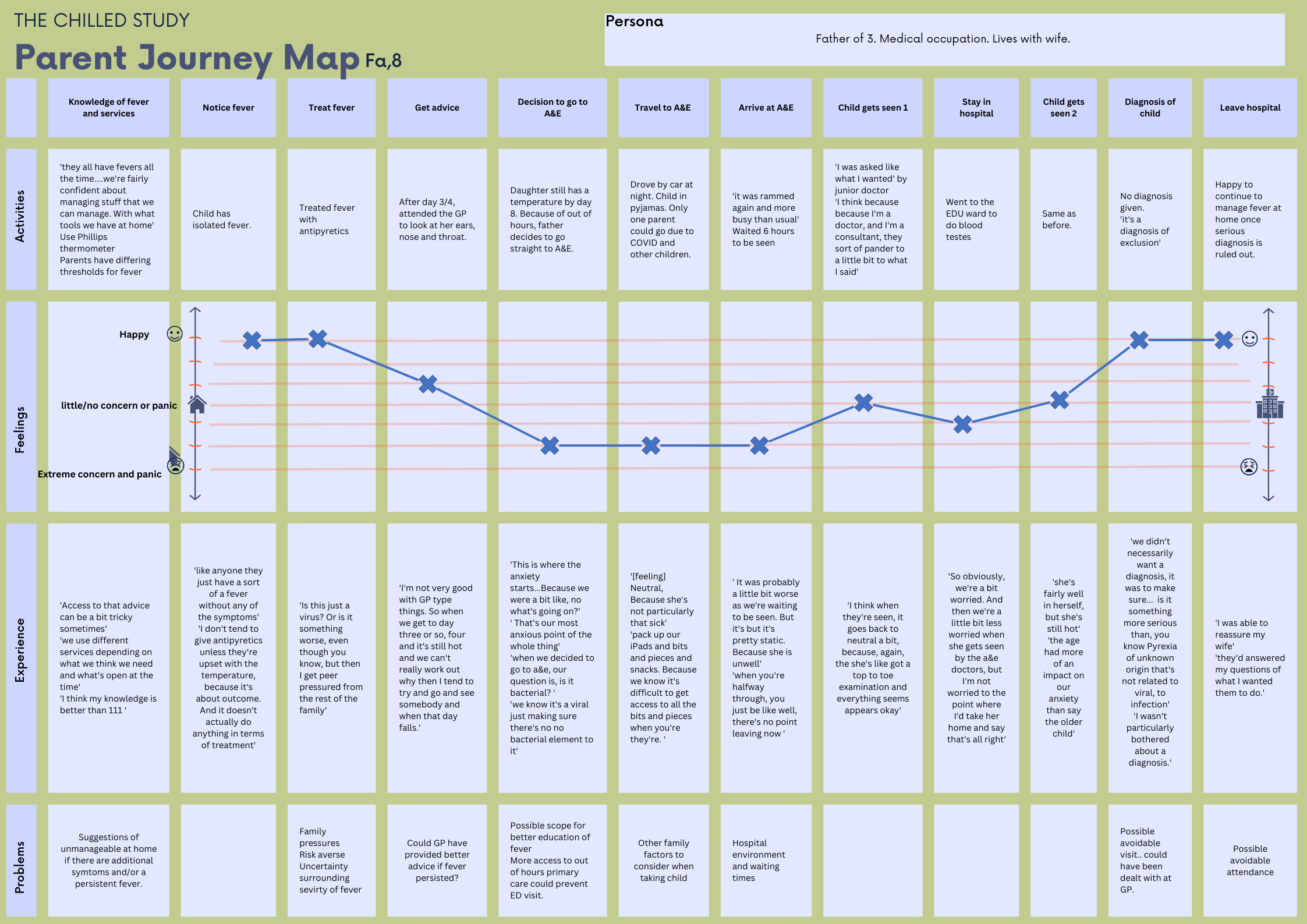

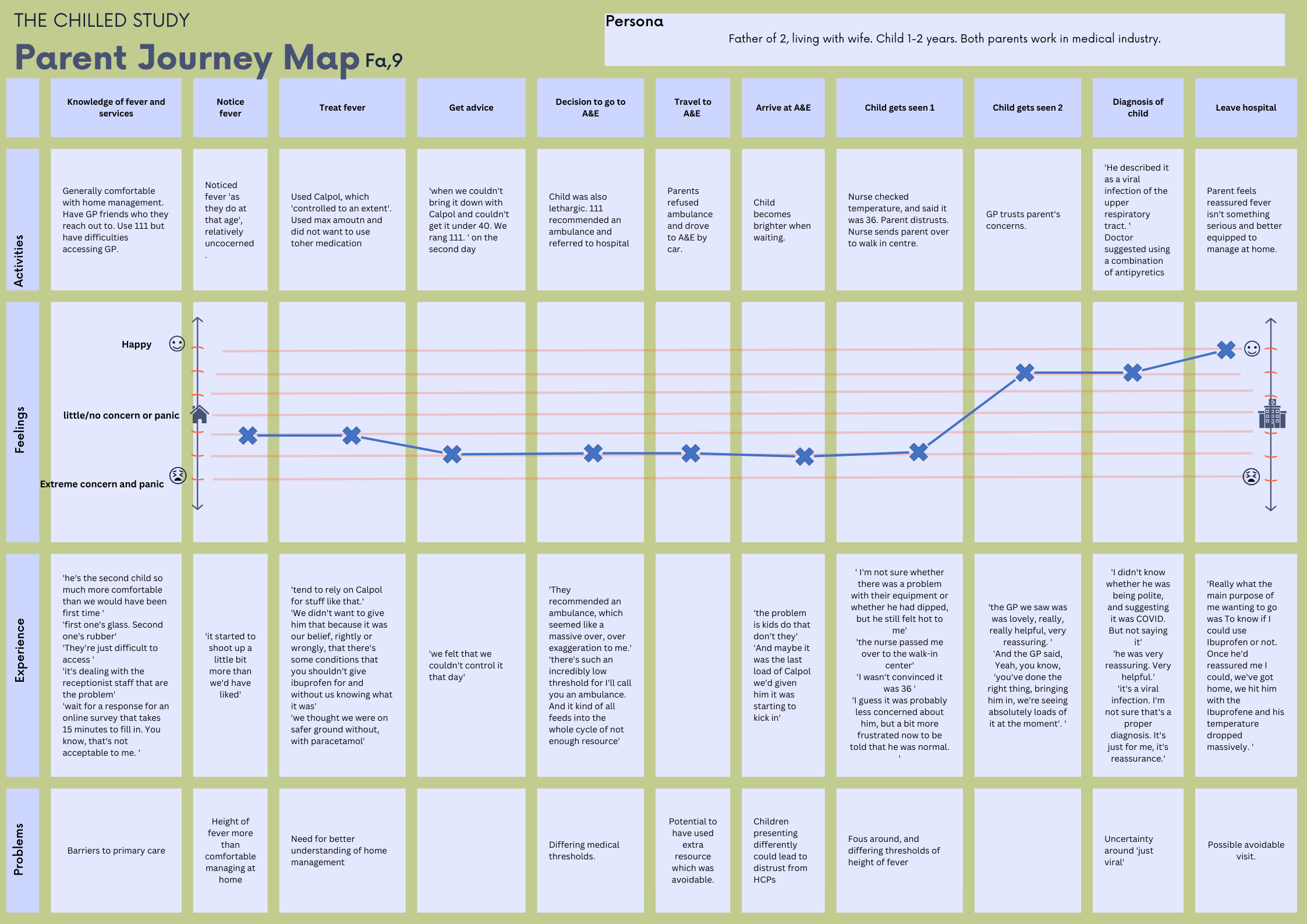

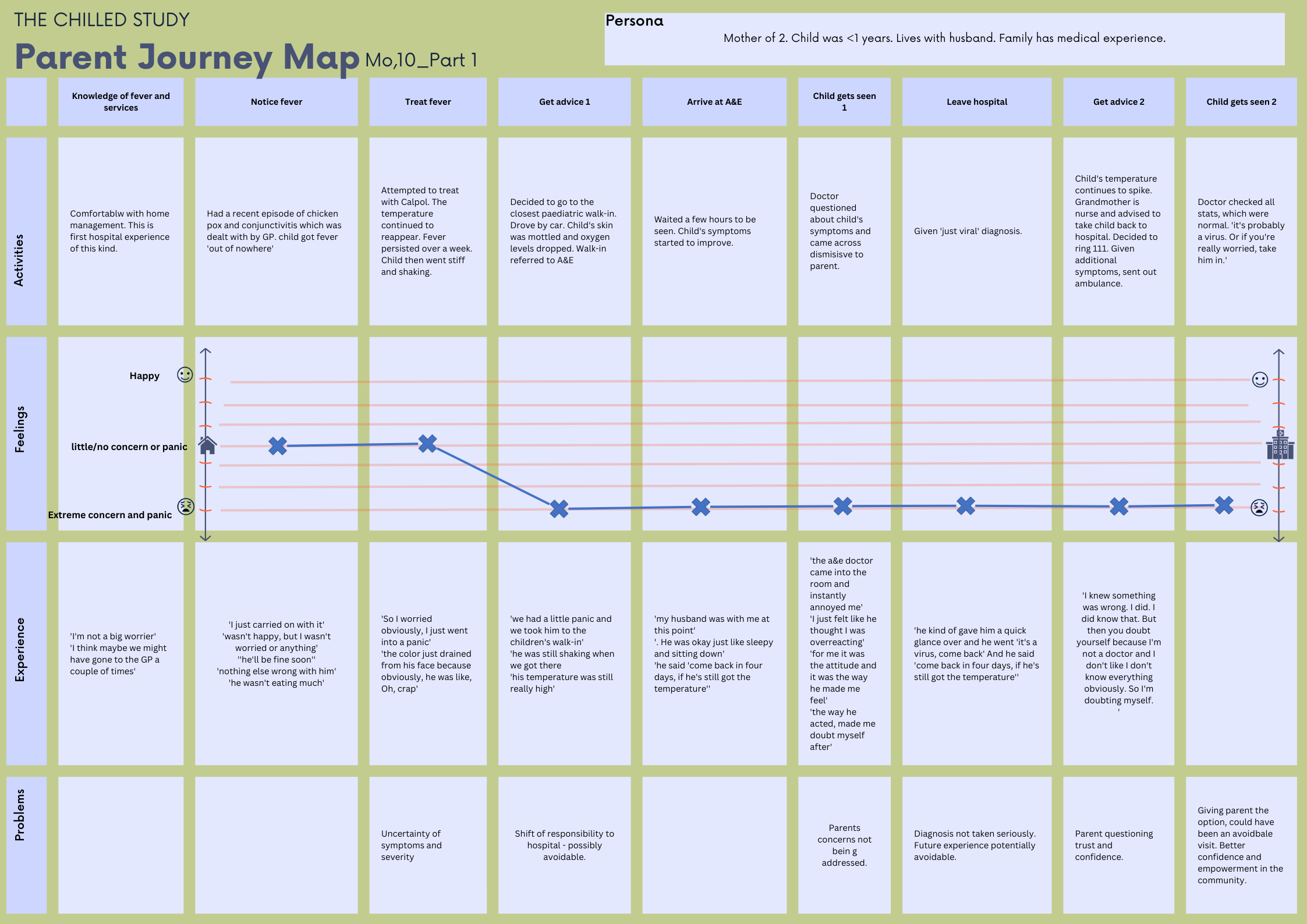

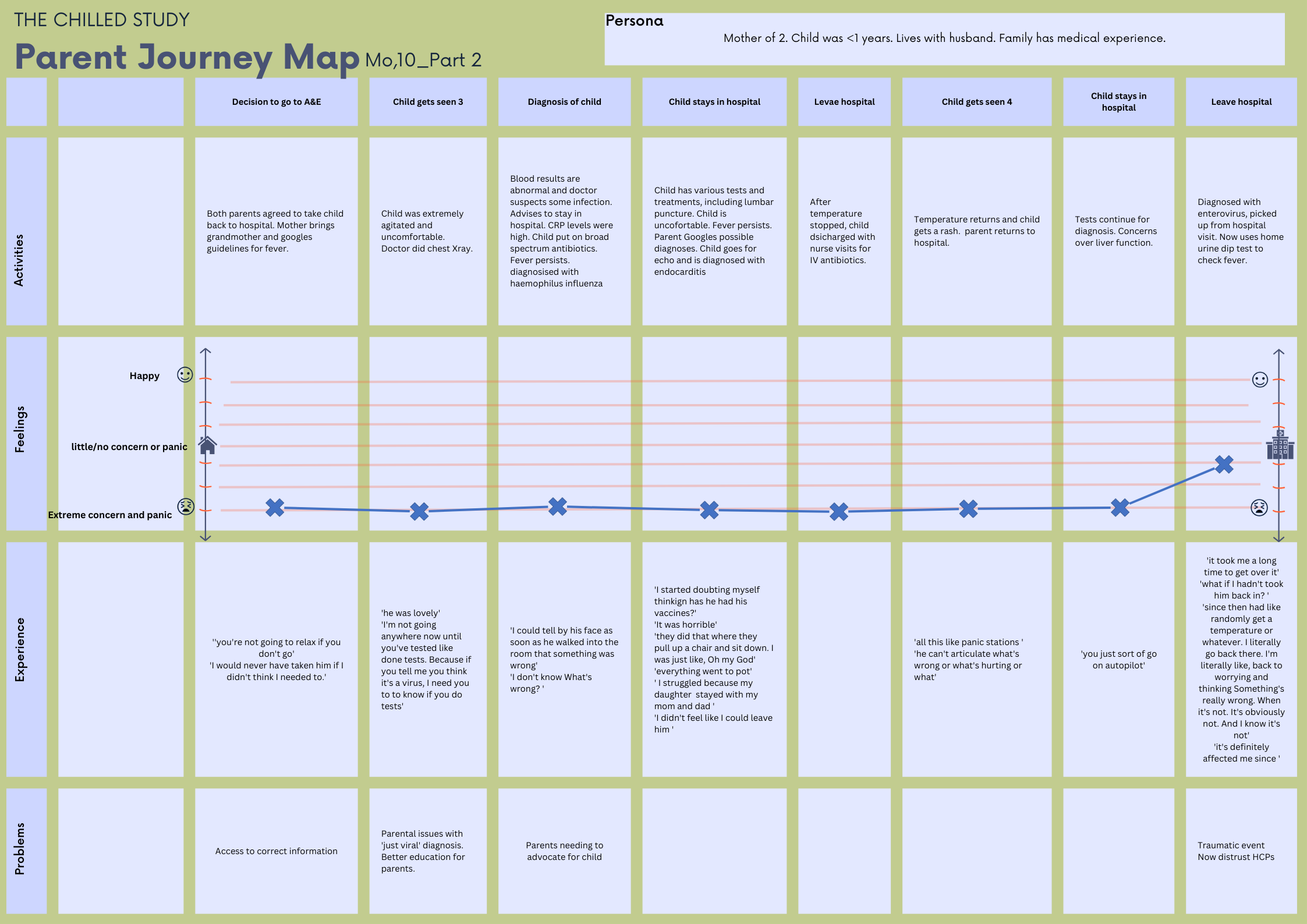

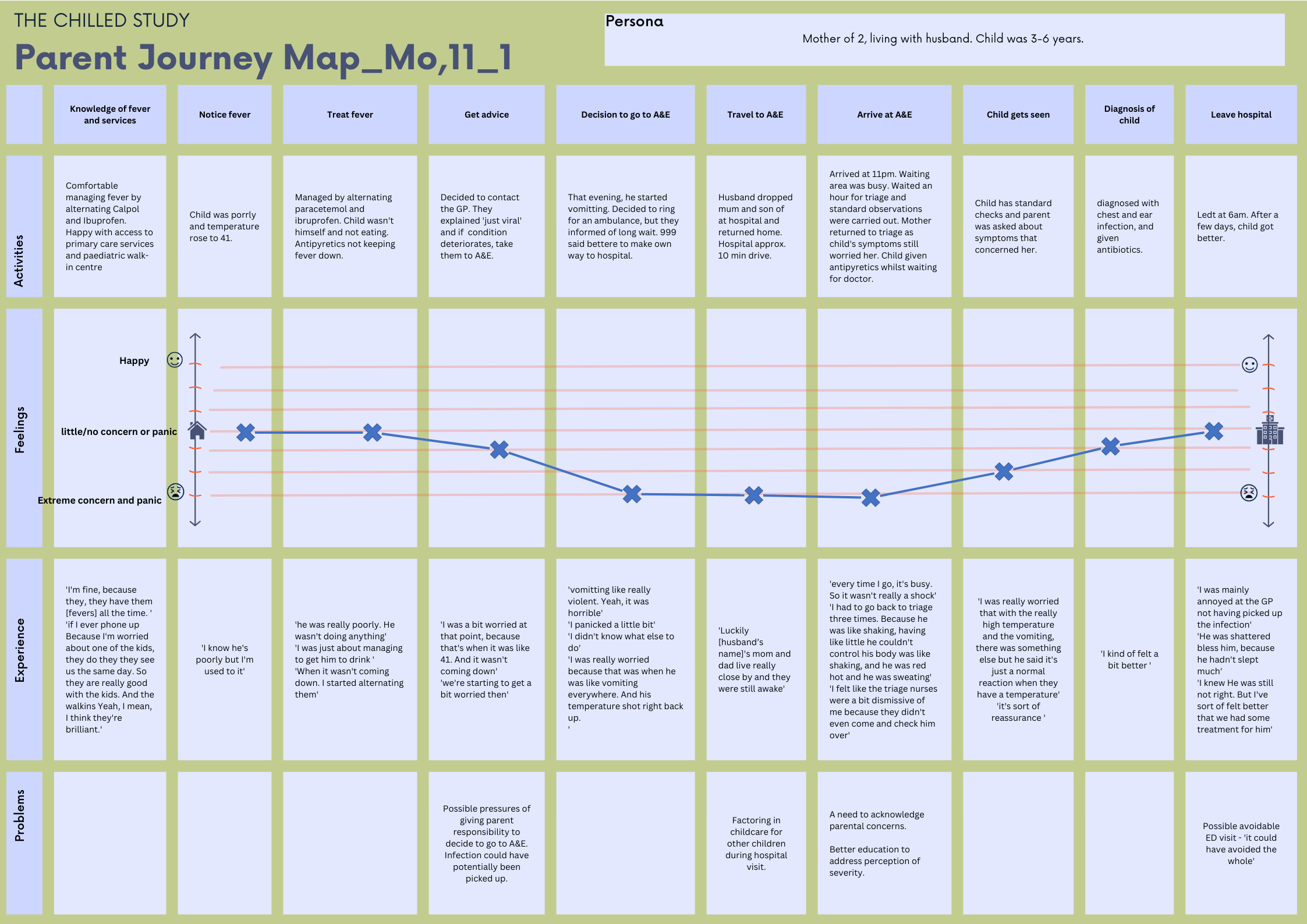

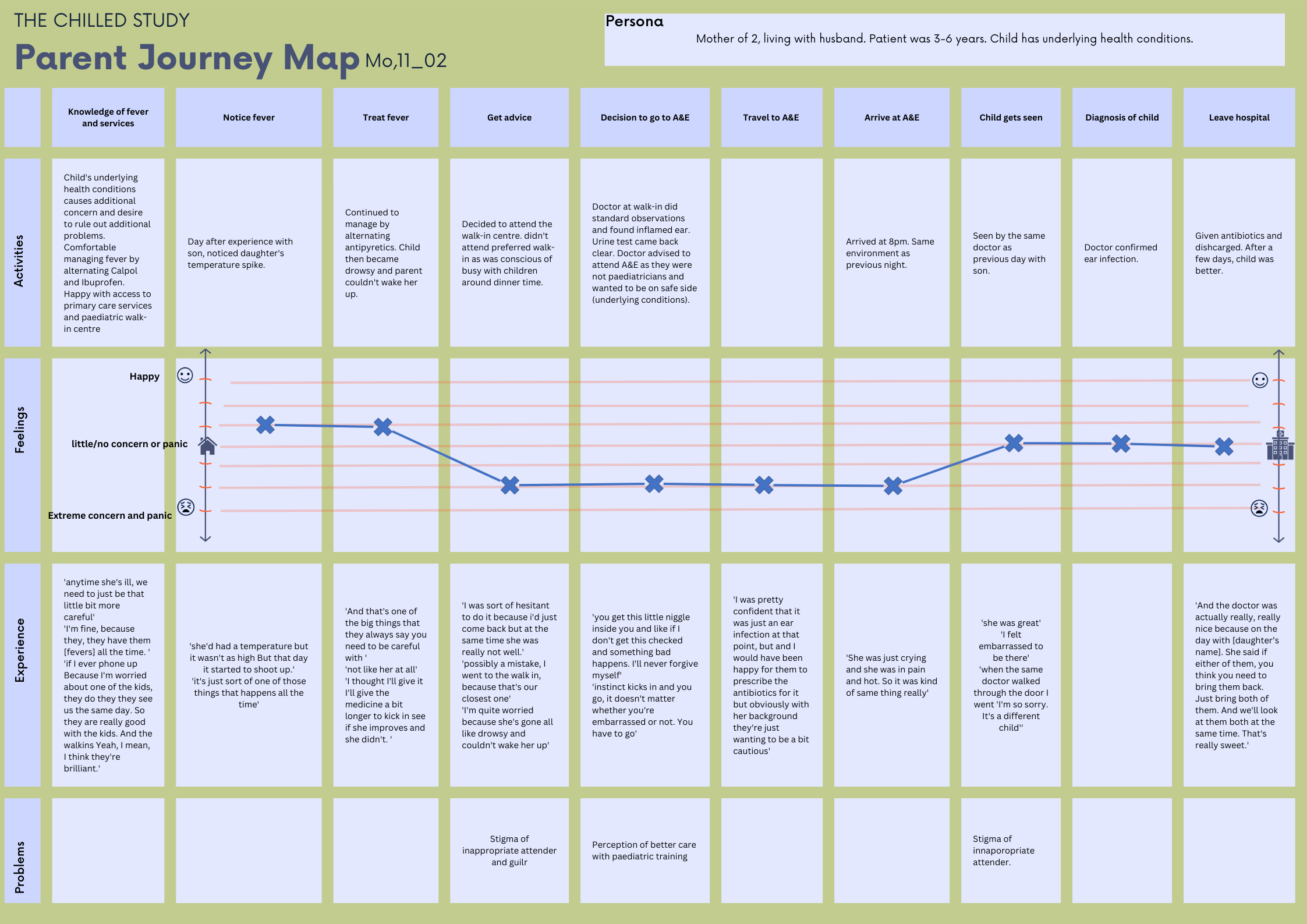

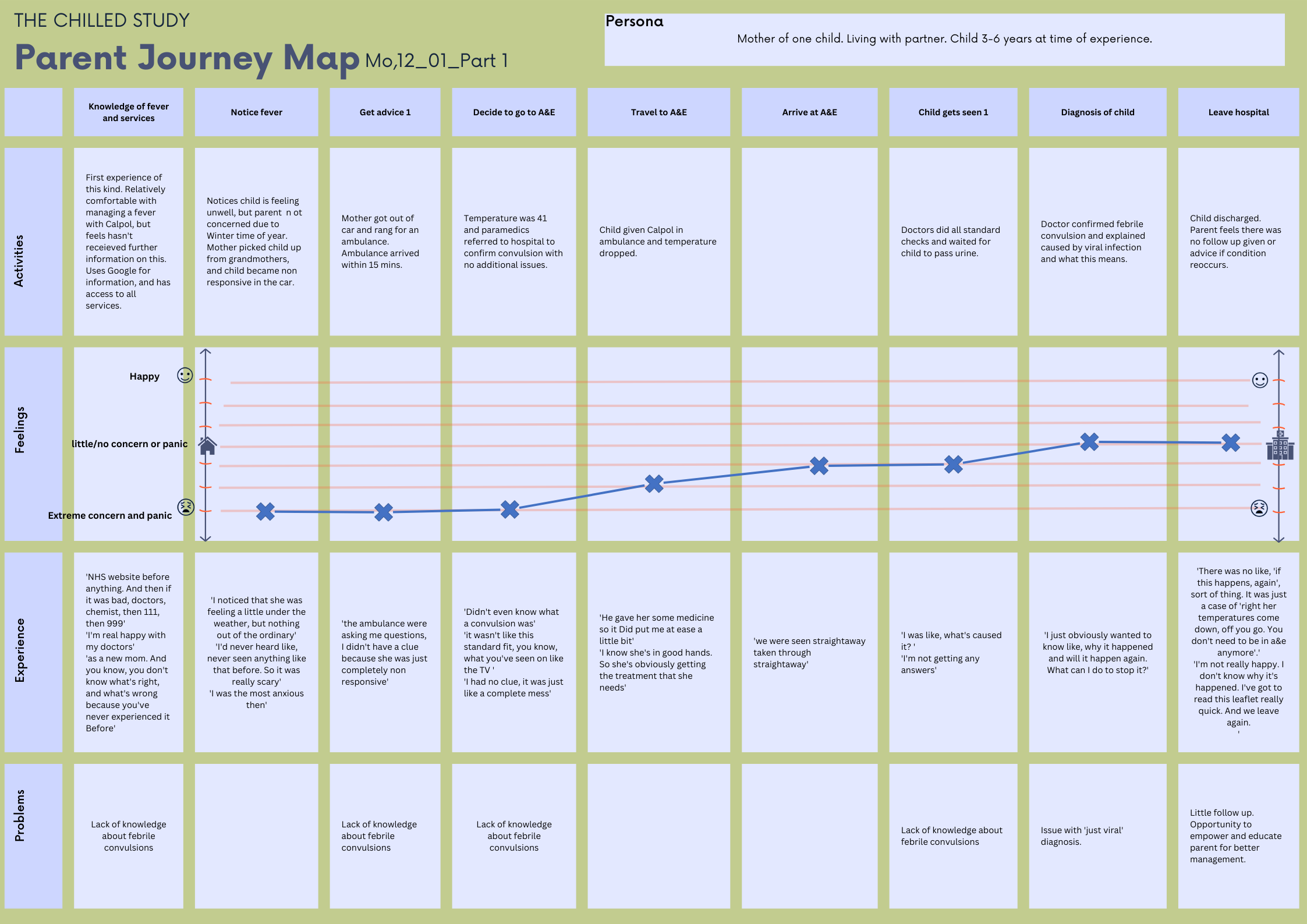

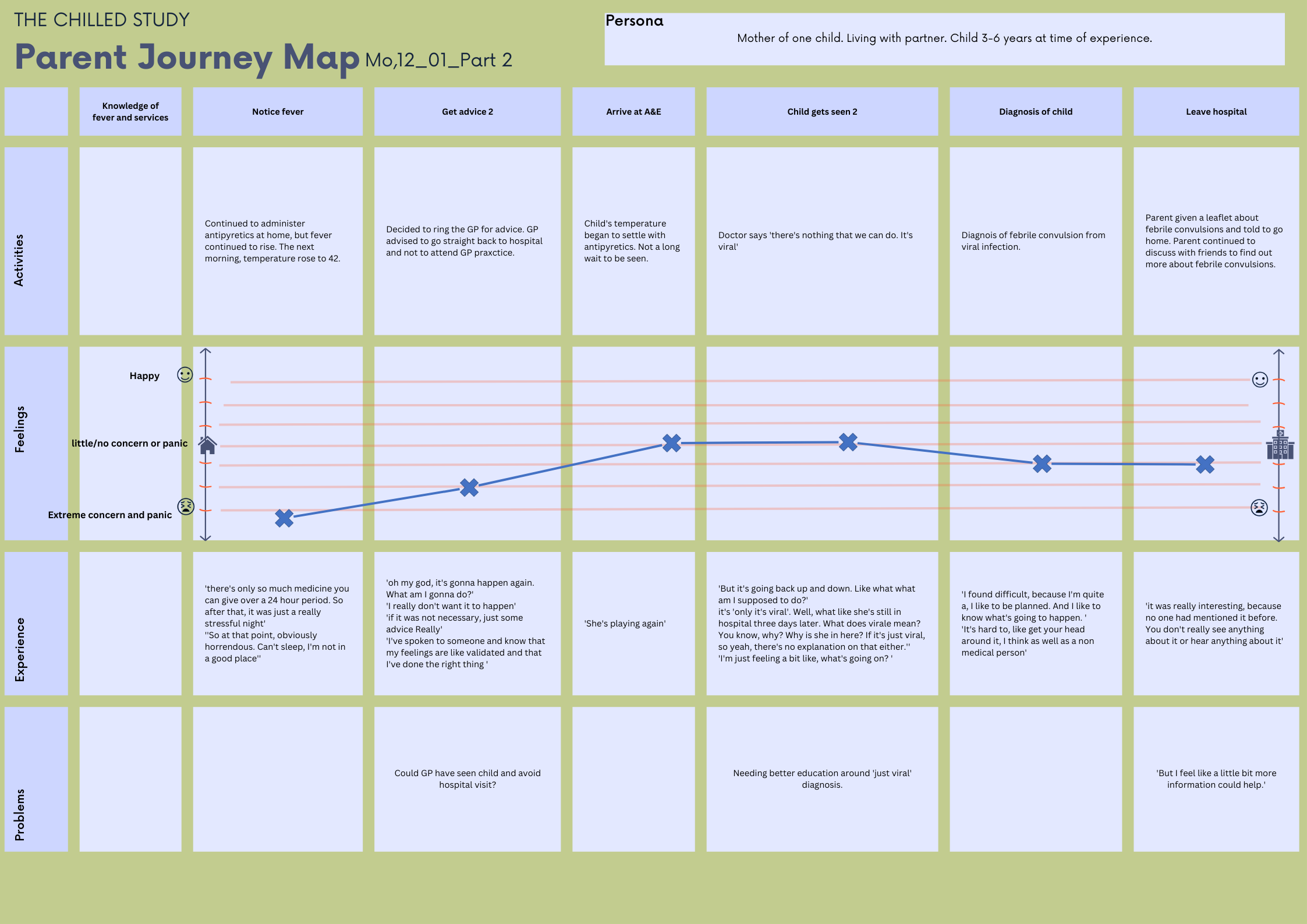

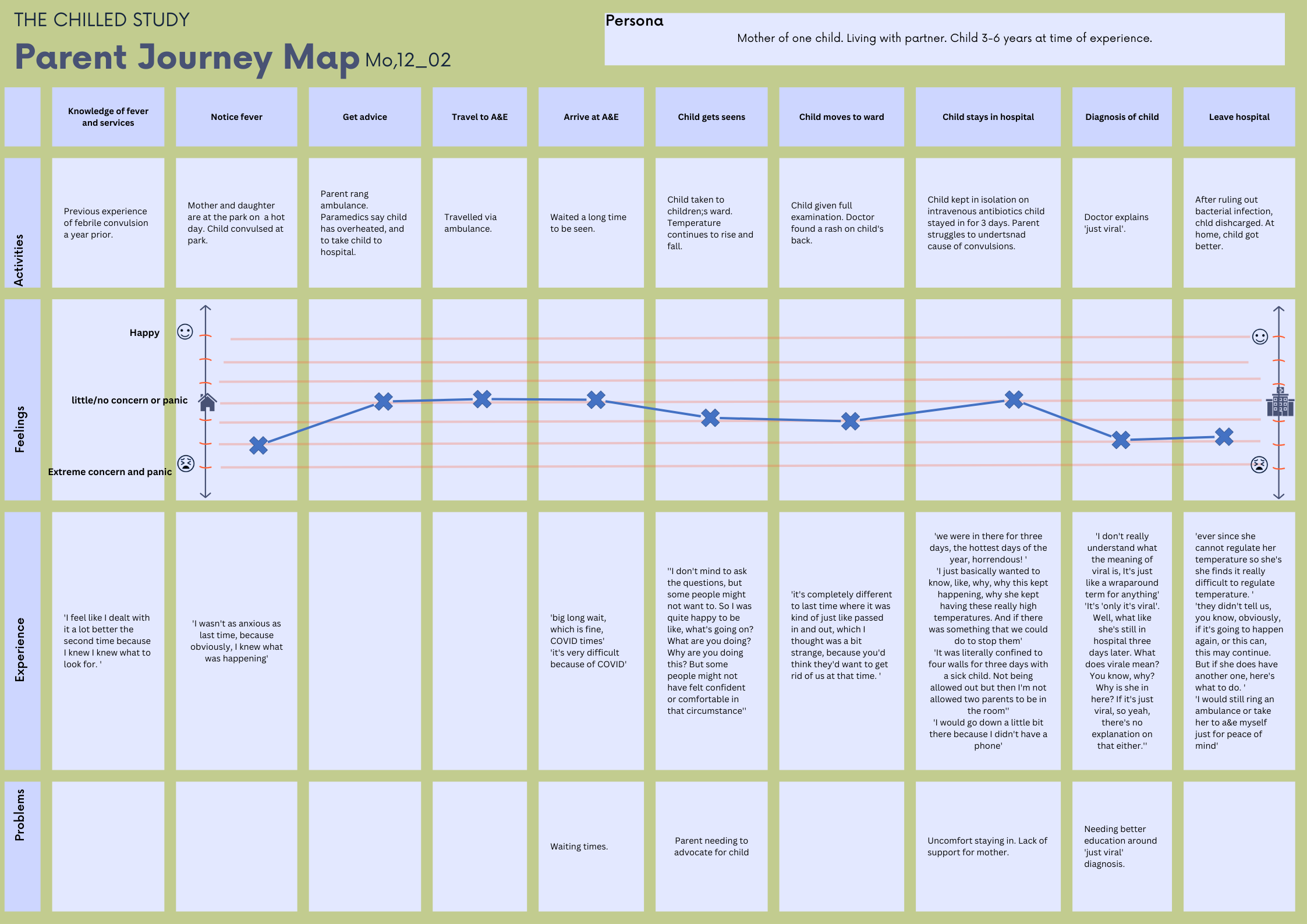

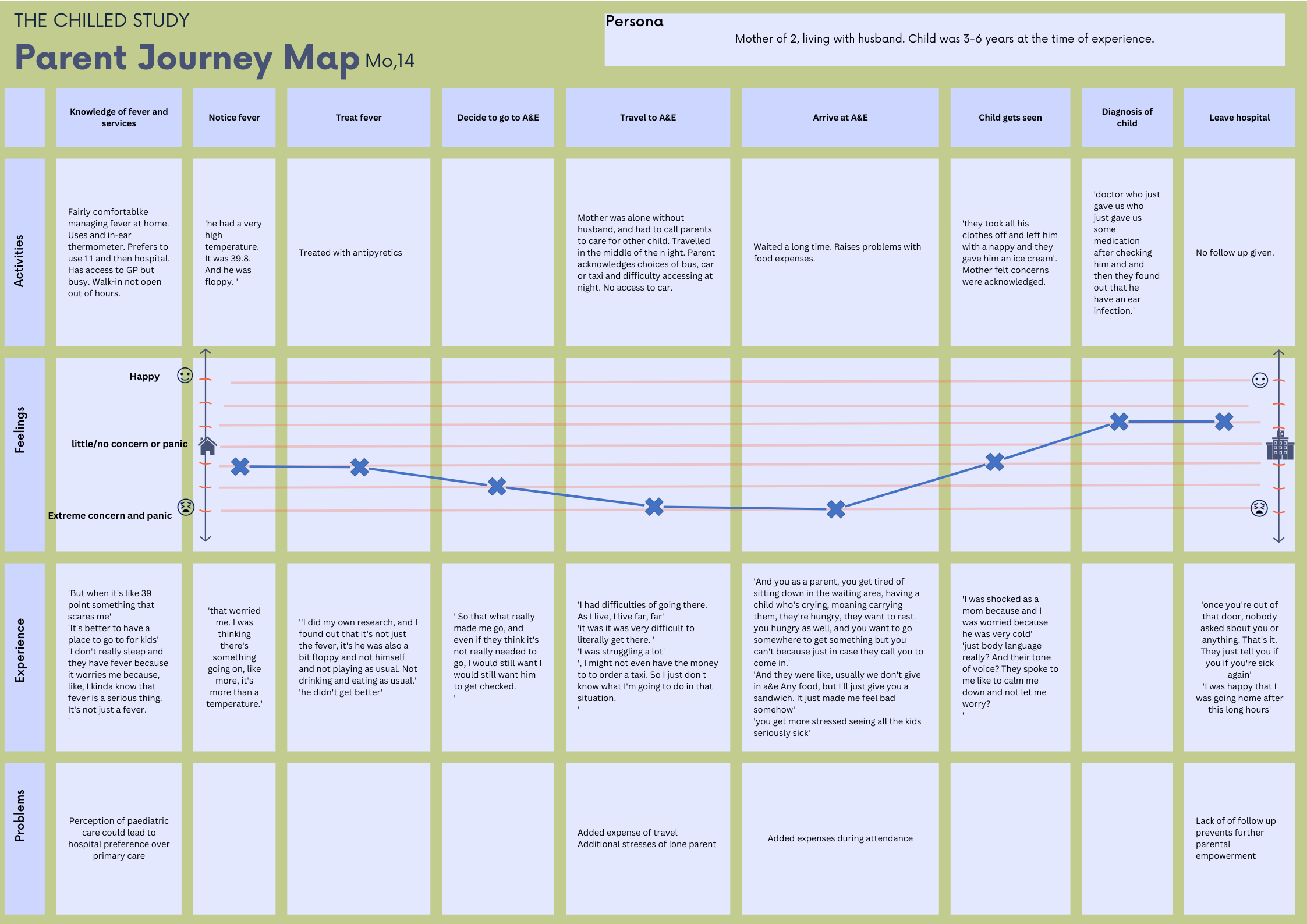


**Parent advert**


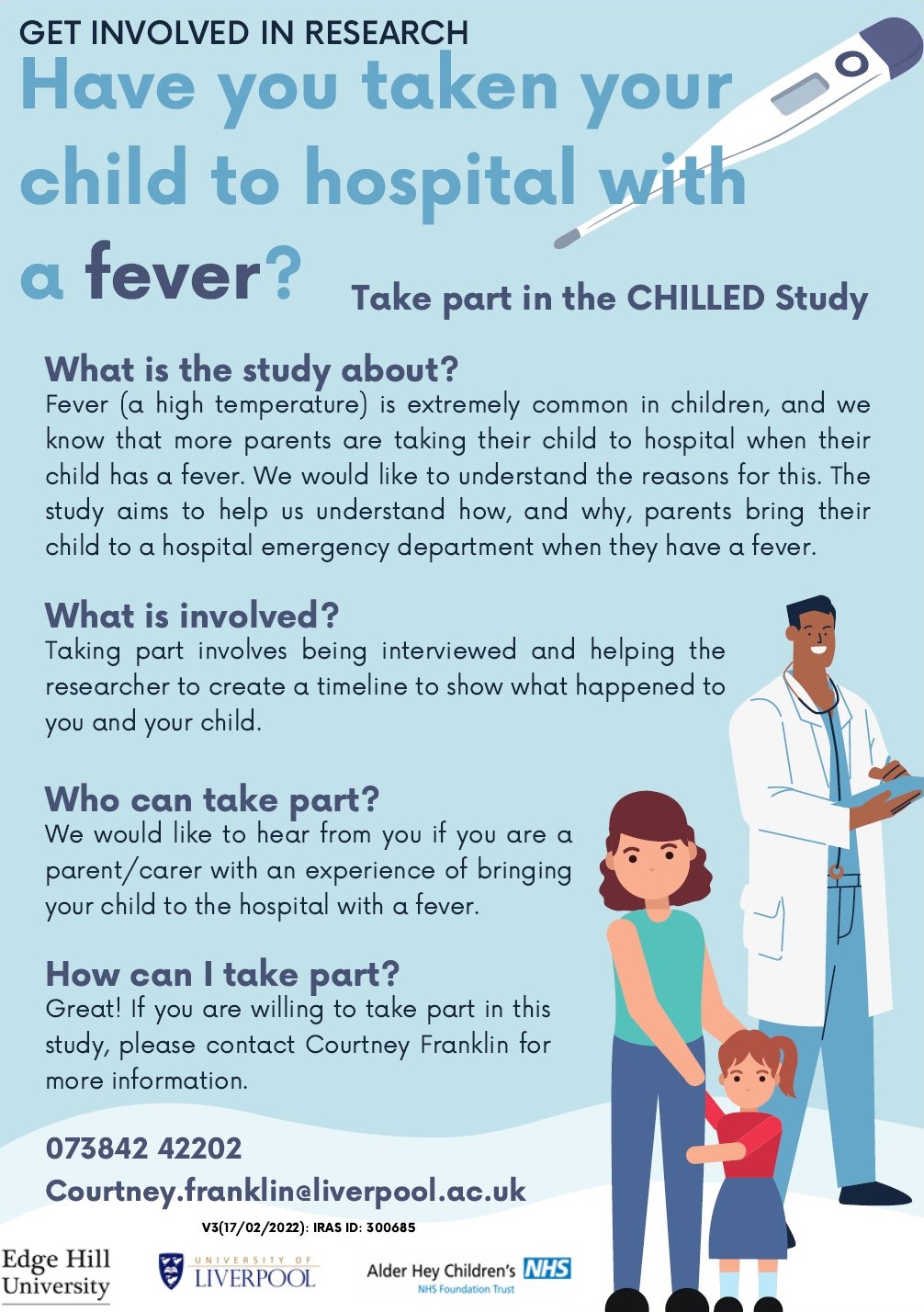


**Parent consent form**


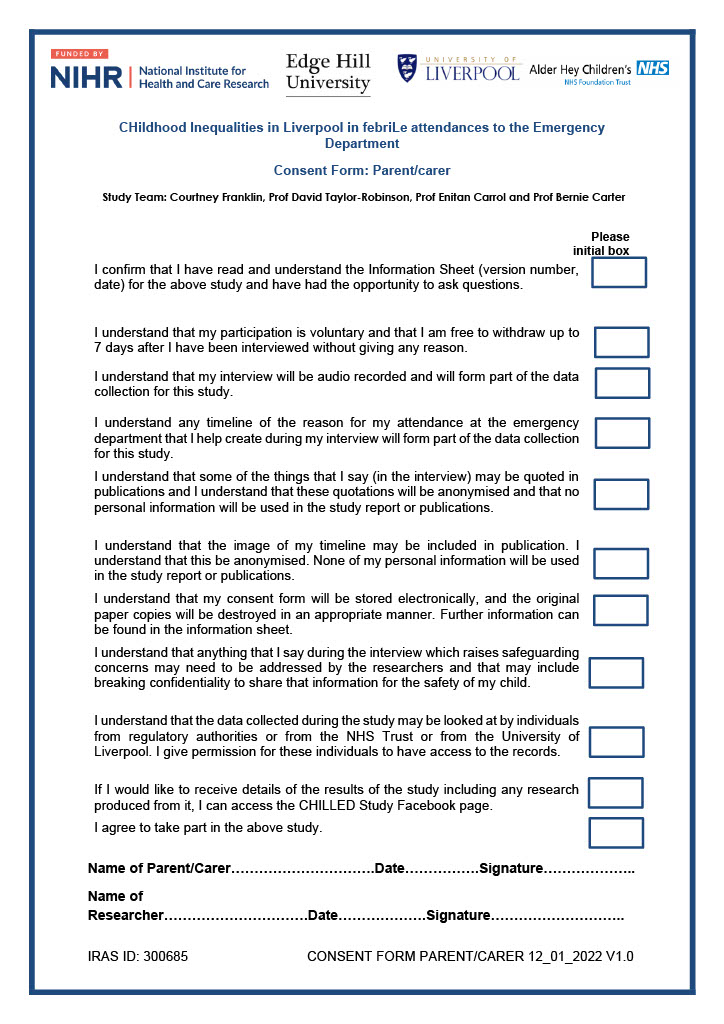


**Parent pre-interview information sheet**
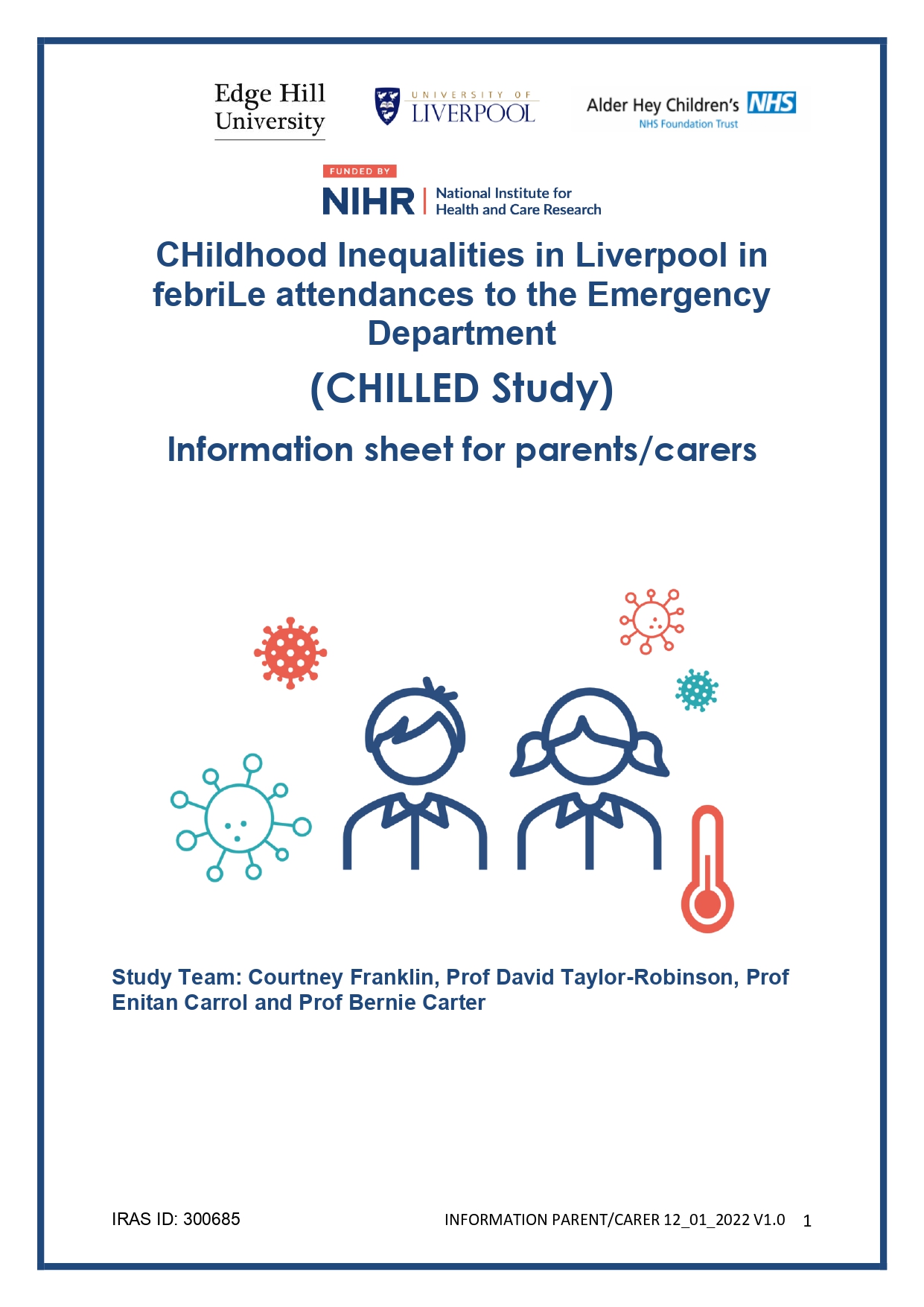


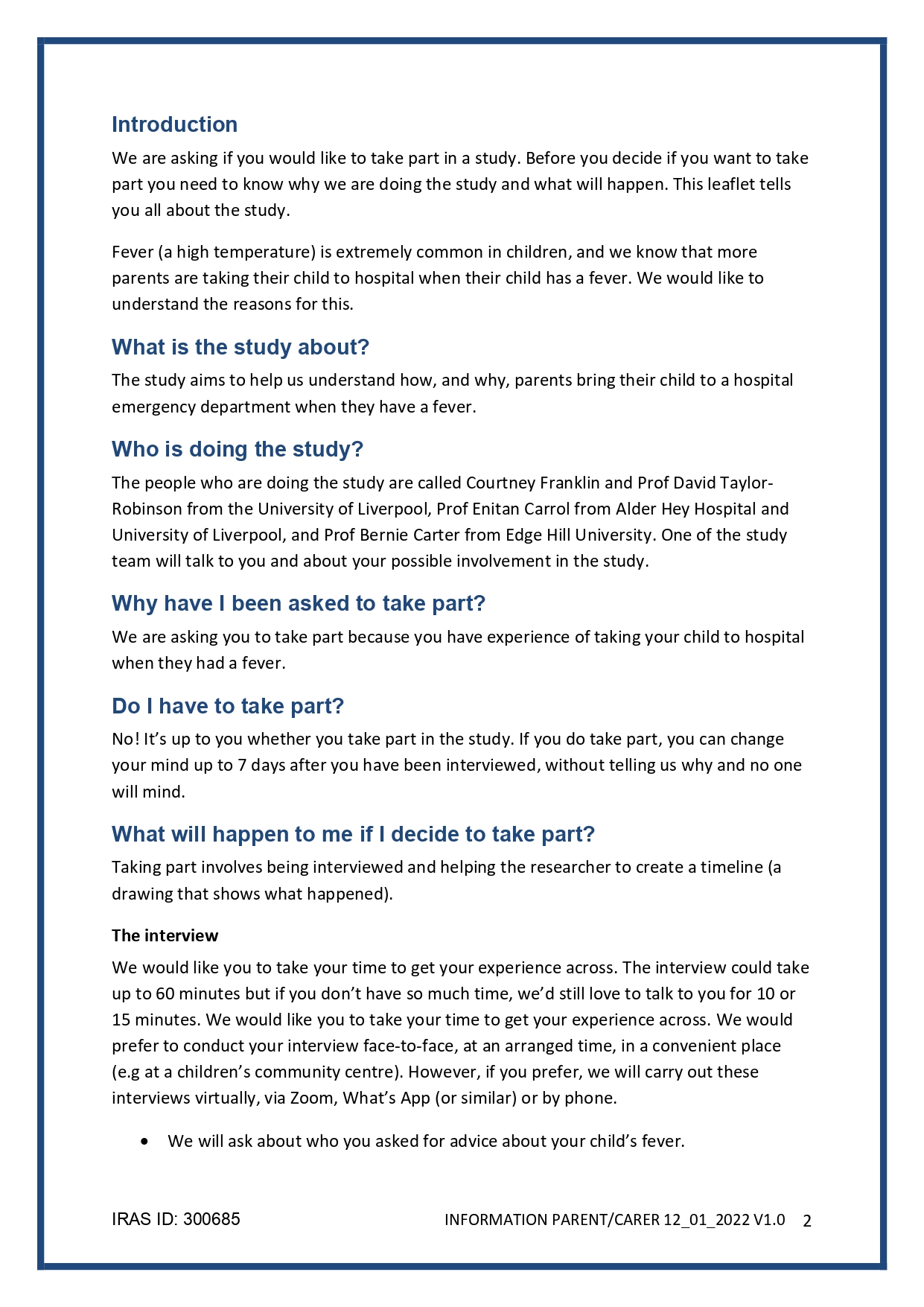

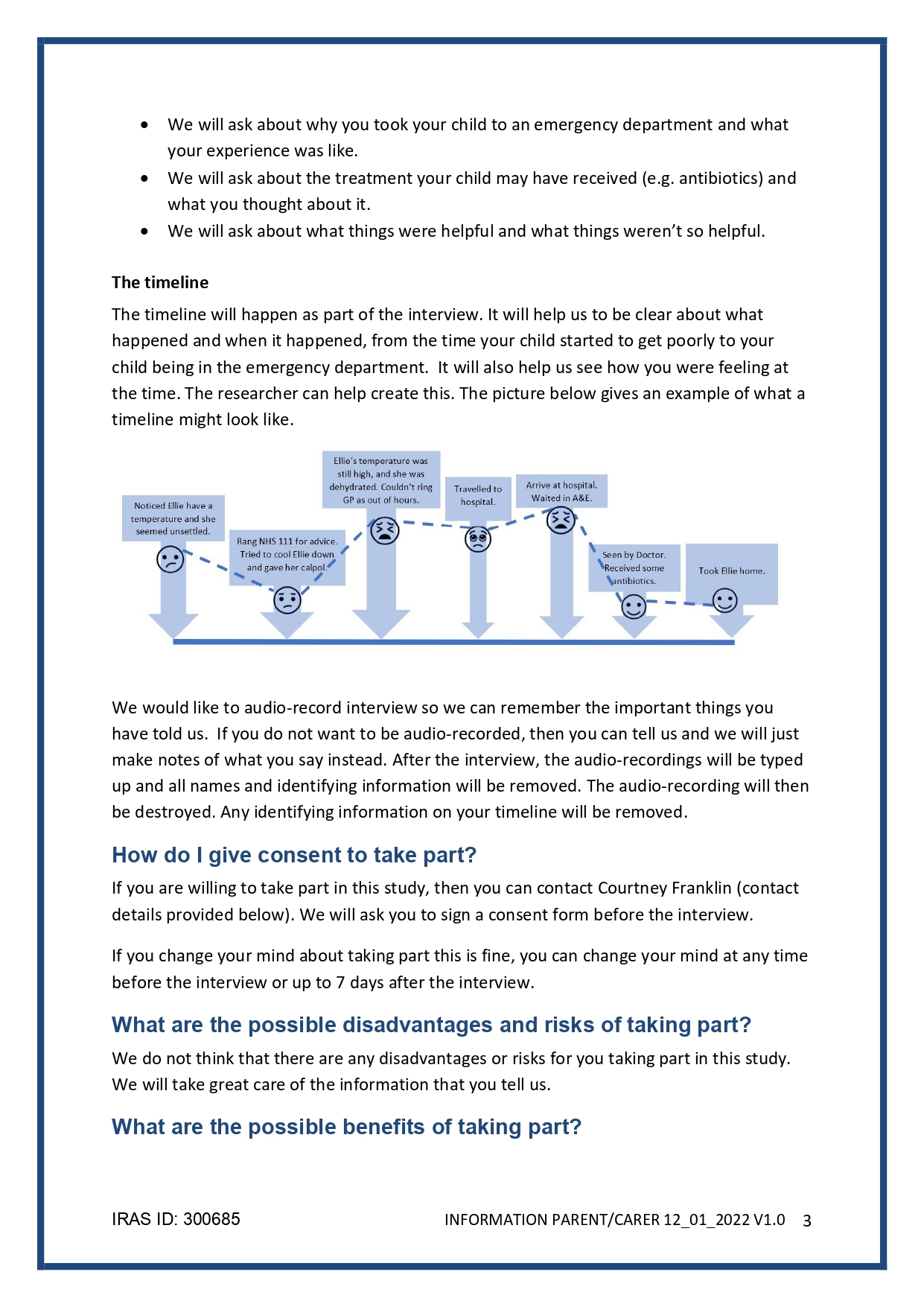

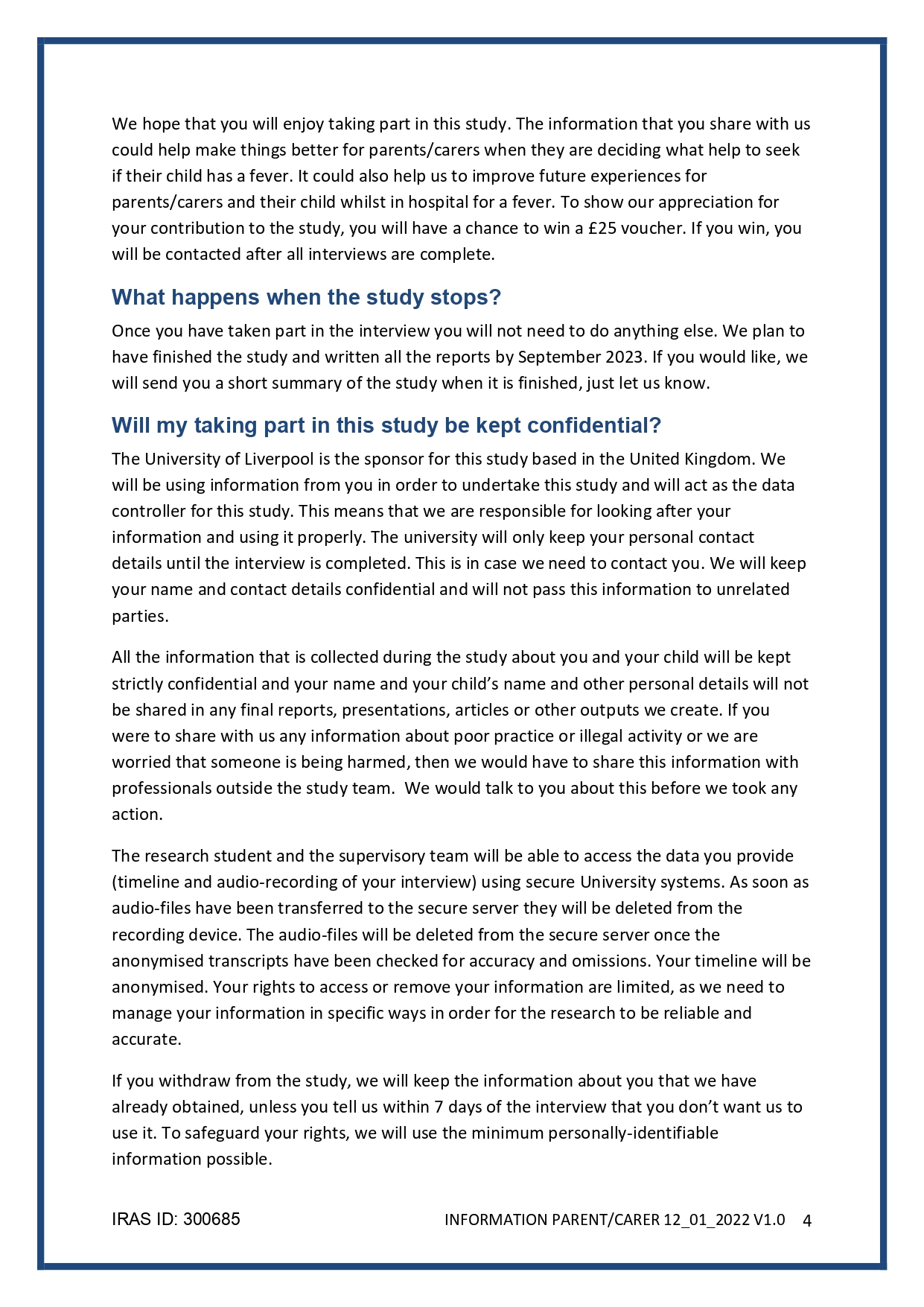

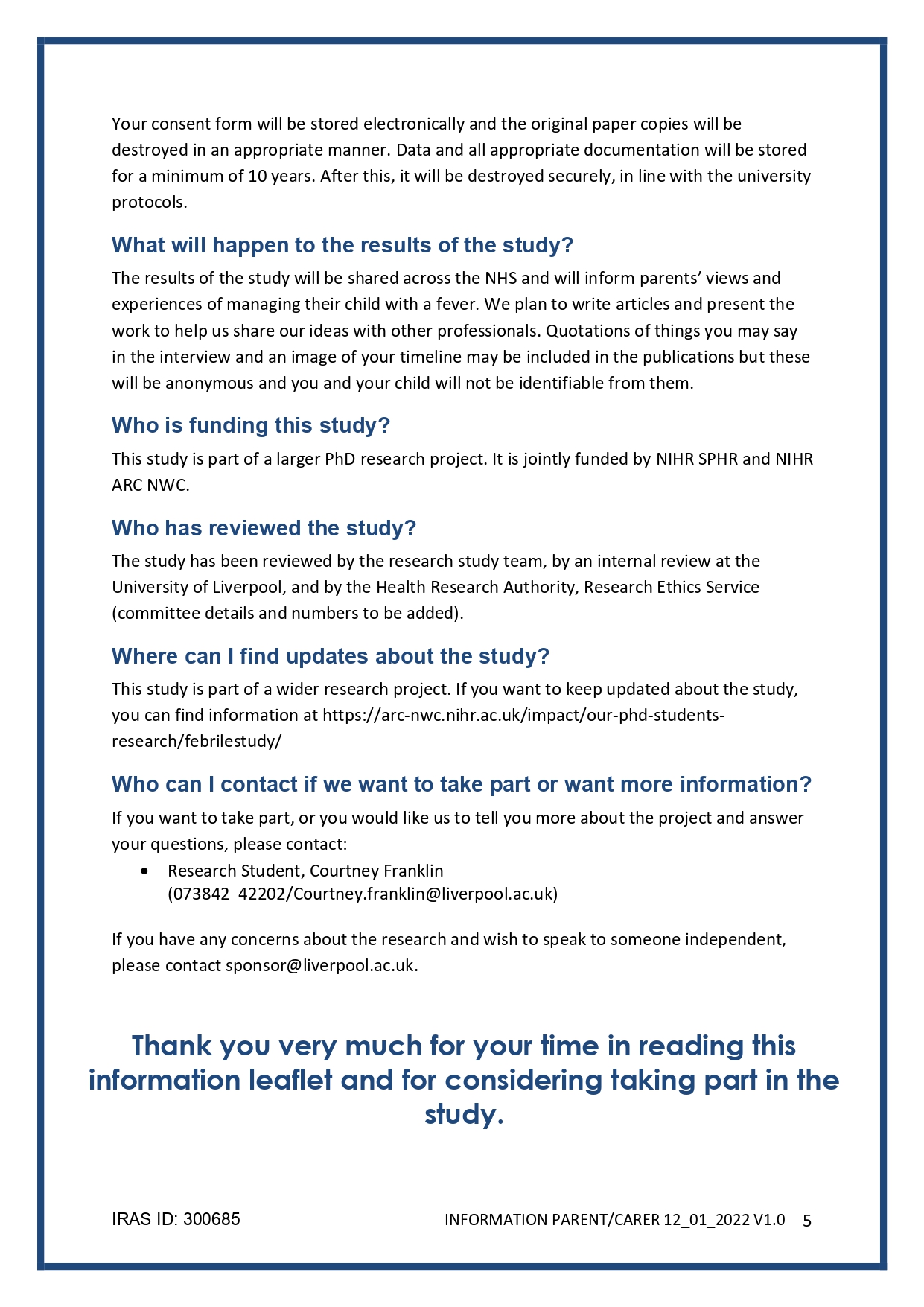


**Parent interview guide**
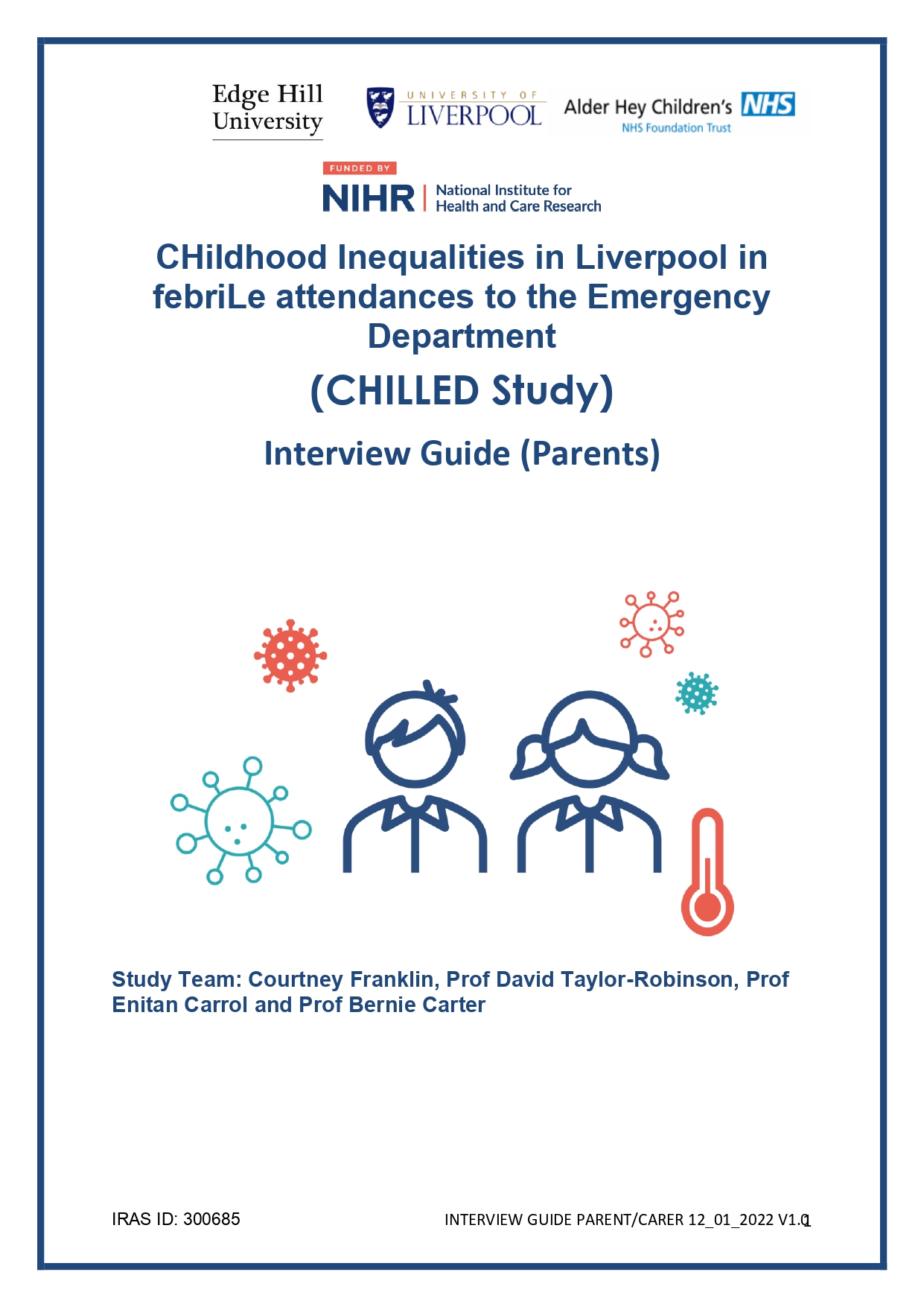

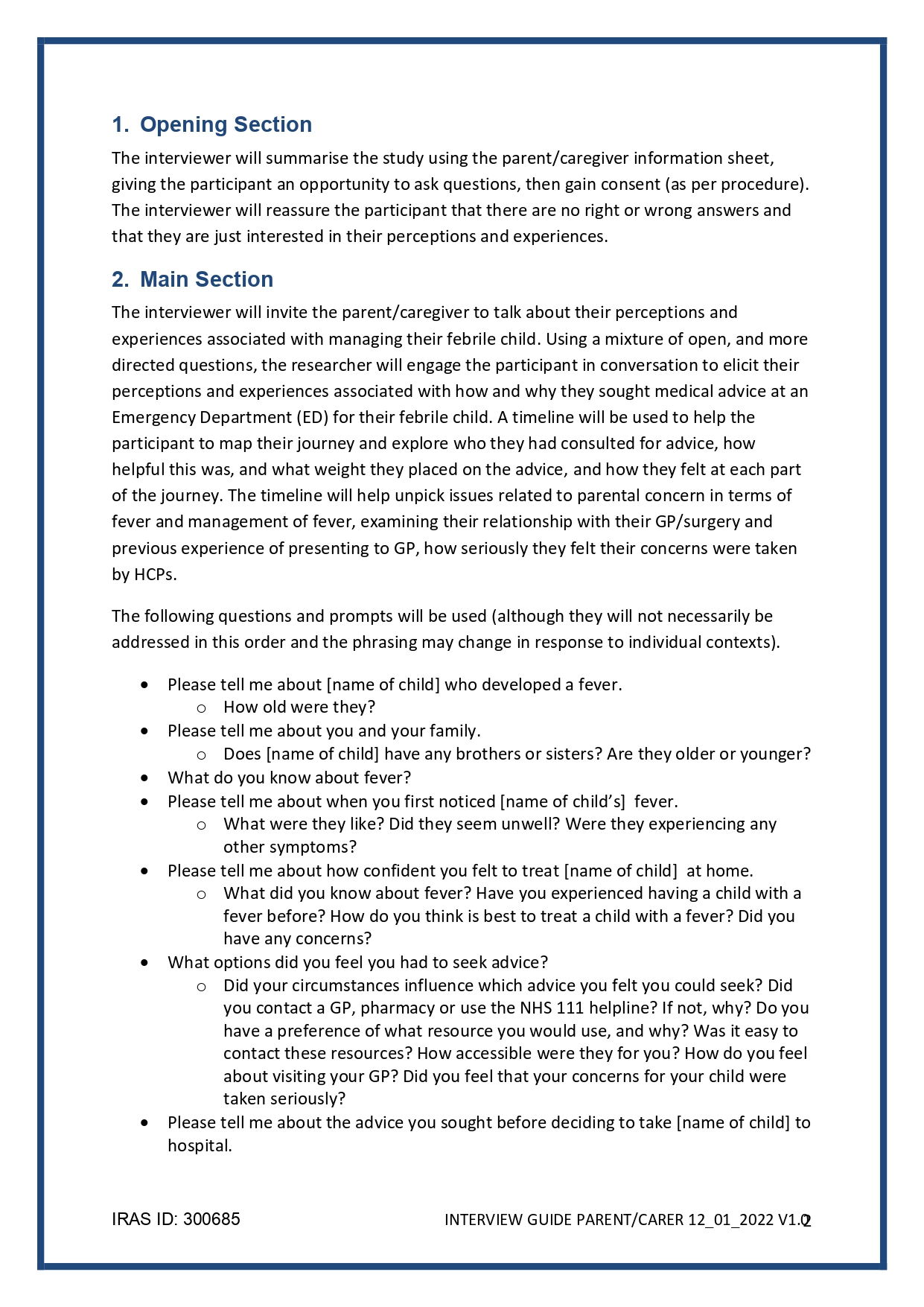

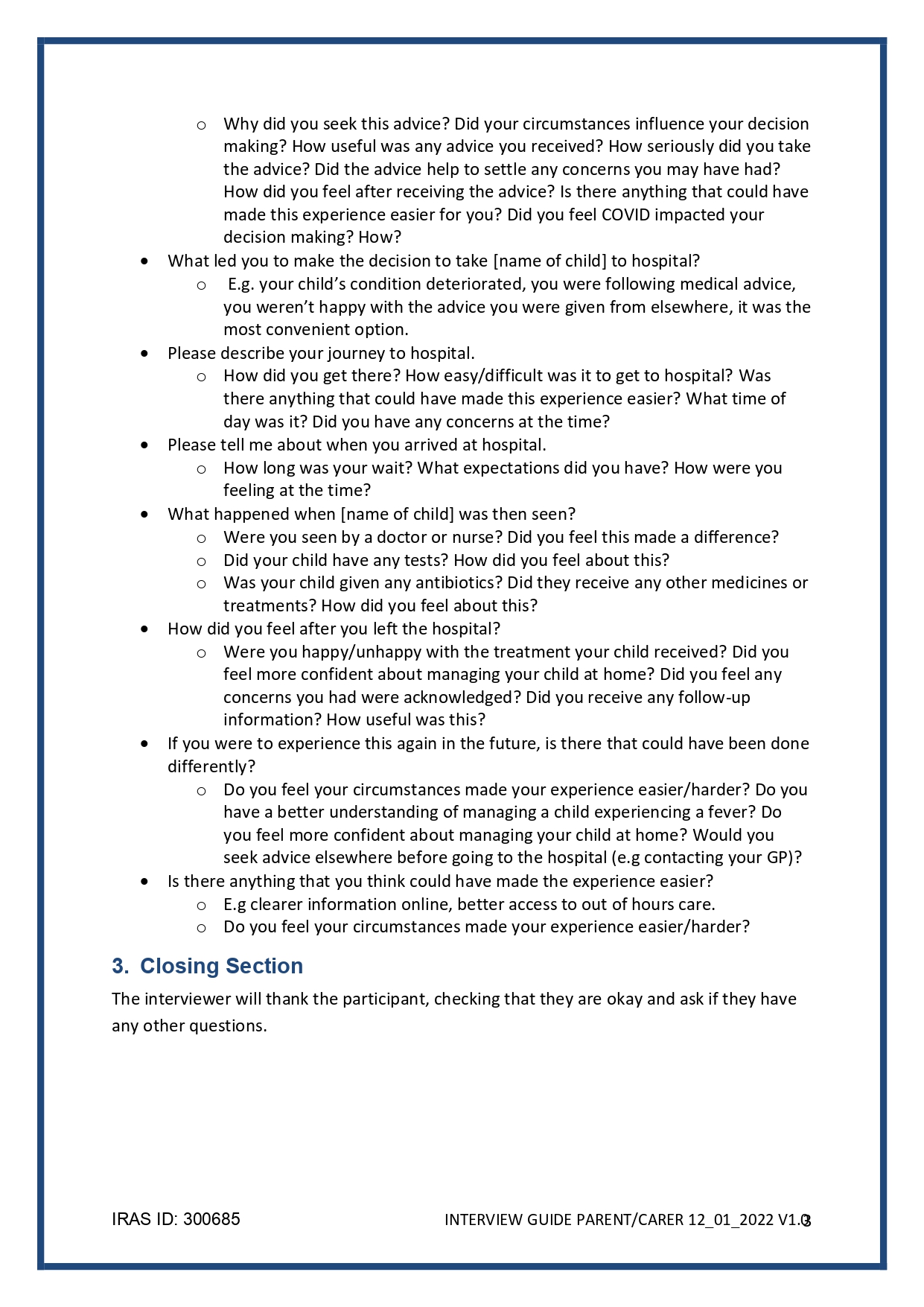


**Parent post-interview information sheet**

**
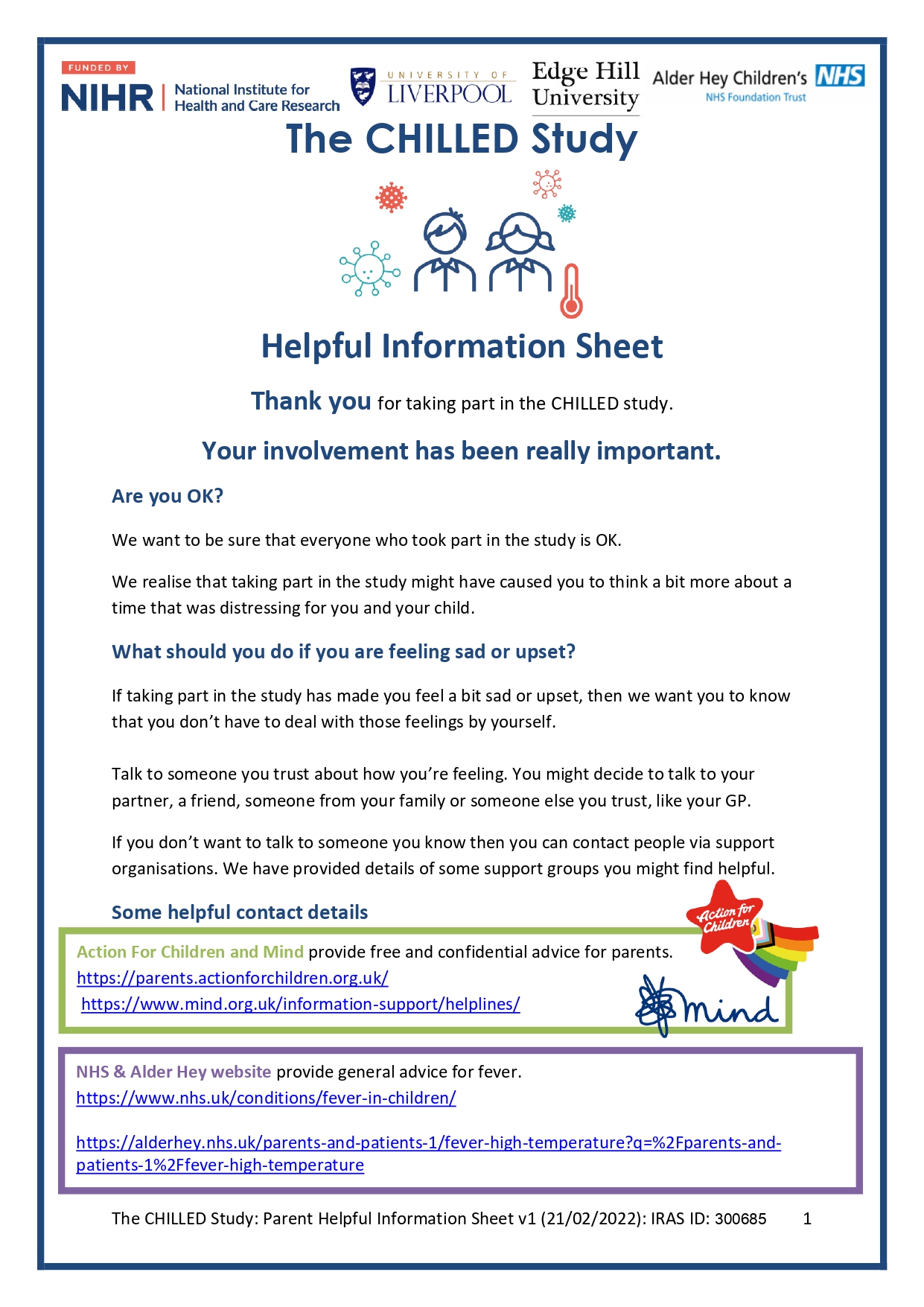
**
